# Aberrant m5C hypermethylation mediates intrinsic resistance to gefitinib through NSUN2/YBX1/QSOX1 axis in EGFR-mutant non-small-cell lung cancer

**DOI:** 10.1186/s12943-023-01780-4

**Published:** 2023-05-09

**Authors:** Yueqin Wang, Jingyao Wei, Luyao Feng, Ouwen Li, Lan Huang, Shaoxuan Zhou, Yingjie Xu, Ke An, Yu Zhang, Ruiying Chen, Lulu He, Qiming Wang, Han Wang, Yue Du, Ruijuan Liu, Chunmin Huang, Xiaojian Zhang, Yun-gui Yang, Quancheng Kan, Xin Tian

**Affiliations:** 1 Department of Pharmacy, the First Affiliated Hospital of Zhengzhou University, No.1 Jianshedong Rd, Zhengzhou, Henan 450052 China; 2 Henan Key Laboratory of Precision Clinical Pharmacy, Zhengzhou University, Zhengzhou, 450052 China; 3 Biotherapy Center, the First Affiliated Hospital of Zhengzhou University, Zhengzhou, Henan 450052 China; 4 Department of Respiratory Medicine, the First Affiliated Hospital of Zhengzhou University, Zhengzhou, 450052 China; 5 Biobank of the First Affiliated Hospital of Zhengzhou University, Zhengzhou, 450052 China; 6 Department of Internal Medicine, Affiliated Cancer Hospital of Zhengzhou University, Zhengzhou, 450052 China; 7 Key Laboratory of Genomic and Precision Medicine, Collaborative Innovation Center of Genetics and Development, Beijing Institute of Genomics, Chinese Academy of Sciences, China National Center for Bioinformation, Beijing, 100101 China

**Keywords:** RNA 5-methylcytosinine, NSUN2, QSOX1, YBX1, Intrinsic resistance, Gefitinib

## Abstract

**Background:**

RNA 5-methylcytosine (m^5^C) modification plays critical roles in the pathogenesis of various tumors. However, the function and molecular mechanism of RNA m^5^C modification in tumor drug resistance remain unclear.

**Methods:**

The correlation between RNA m^5^C methylation, m^5^C writer NOP2/Sun RNA methyltransferase family member 2 (NSUN2) and EGFR-TKIs resistance was determined in non-small-cell lung cancer (NSCLC) cell lines and patient samples. The effects of NSUN2 on EGFR-TKIs resistance were investigated by gain- and loss-of-function assays *in vitro* and *in vivo*. RNA-sequencing (RNA-seq), RNA bisulfite sequencing (RNA-BisSeq) and m^5^C methylated RNA immunoprecipitation-qPCR (MeRIP-qPCR) were performed to identify the target gene of NSUN2 involved in EGFR-TKIs resistance. Furthermore, the regulatory mechanism of NSUN2 modulating the target gene expression was investigated by functional rescue and puromycin incorporation assays.

**Results:**

RNA m^5^C hypermethylation and NSUN2 were significantly correlated with intrinsic resistance to EGFR-TKIs. Overexpression of NSUN2 resulted in gefitinib resistance and tumor recurrence, while genetic inhibition of NSUN2 led to tumor regression and overcame intrinsic resistance to gefitinib *in vitro* and *in vivo*. Integrated RNA-seq and m^5^C-BisSeq analyses identified quiescin sulfhydryl oxidase 1 (QSOX1) as a potential target of aberrant m^5^C modification. NSUN2 methylated QSOX1 coding sequence region, leading to enhanced QSOX1 translation through m^5^C reader Y-box binding protein 1 (YBX1).

**Conclusions:**

Our study reveals a critical function of aberrant RNA m^5^C modification via the NSUN2-YBX1-QSOX1 axis in mediating intrinsic resistance to gefitinib in EGFR-mutant NSCLC.

**Supplementary Information:**

The online version contains supplementary material available at 10.1186/s12943-023-01780-4.

## Introduction

Intrinsic and acquired drug resistance to EGFR tyrosine kinase inhibitors (EGFR-TKIs) is the main cause of treatment failure in patients with EGFR-mutant non-small-cell lung cancer (NSCLC) [1–3]. Molecular mechanisms of acquired resistance to EGFR-TKIs have been well elucidated, including secondary T790M mutation (50–60%), MET amplification (5–22%), HER2 amplification (~ 12%) and transformation to small cell lung cancer (SCLC) (3–10%) [4–8]. However, the mechanism underlying intrinsic resistance to EGFR-TKIs remains largely unknown. Although EGFR exon 20 insertion mutations [9], BIM deletion polymorphism [10, 11], and MDM2 amplification [12] have been identified as causes of intrinsic resistance to EGFR-TKIs in certain cases, the actual molecular mechanism appears to be much more complex [13] and needs to be addressed urgently to improve patient outcomes.

5-methylcytosine (m^5^C) is a crucial post-transcriptional modification on mammalian mRNA [14, 15]. It is catalyzed by NOP2/Sun domain (NSUN) RNA methyltransferase or DNA methyltransferase 2 (DNMT2) [16] and can be demethylated by tet methylcytosine dioxygenase 2 (TET2) [17]. Aly/REF export factor (ALYREF) recognizes m^5^C-modified mRNA to facilitate mRNA nuclear export, while Y-box binding protein 1 (YBX1) binds directly to m^5^C methylated mRNA to stabilize mRNA [18, 19]. Recently, aberrant mRNA m^5^C modification has been reported to be involved in the pathogenesis and development of bladder cancer, gastric cancer, and esophageal squamous cell carcinoma [18, 20, 21]. However, little attention has been paid to examining the involvement and mechanisms underlying RNA m^5^C modification in tumor drug resistance. Another RNA methylation modification, N6-methyladenosine (m^6^A), has been reported to be involved in acquired resistance to different cancer treatments [22]. Based on all the information above, we hypothesize that NSCLC patients carrying EGFR activating mutations produce aberrant m^5^C methylation upon EGFR-TKIs treatment, leading to intrinsic resistance to EGFR-TKIs.

Here, we report that aberrant m^5^C hypermethylation confers intrinsic resistance to gefitinib by targeting quiescin sulfhydryl oxidase 1 (QSOX1) in NSCLC. RNA m^5^C writer NSUN2 regulates QSOX1 mRNA translation in an m^5^C-YBX1-dependent manner. Genetic silencing of NSUN2-YBX1-QSOX1 pathway overcomes intrinsic gefitinib resistance in NSCLC. Thus, our work reveals a previously unrecognized axis NSUN2-YBX1-QSOX1 signaling in the prognosis and treatment of NSCLC patients with intrinsic resistance to EGFR-TKIs.

## Results

### m^5^C hypermethylation and NSUN2 are correlated with intrinsic gefitinib resistance

**Fig. 1:**
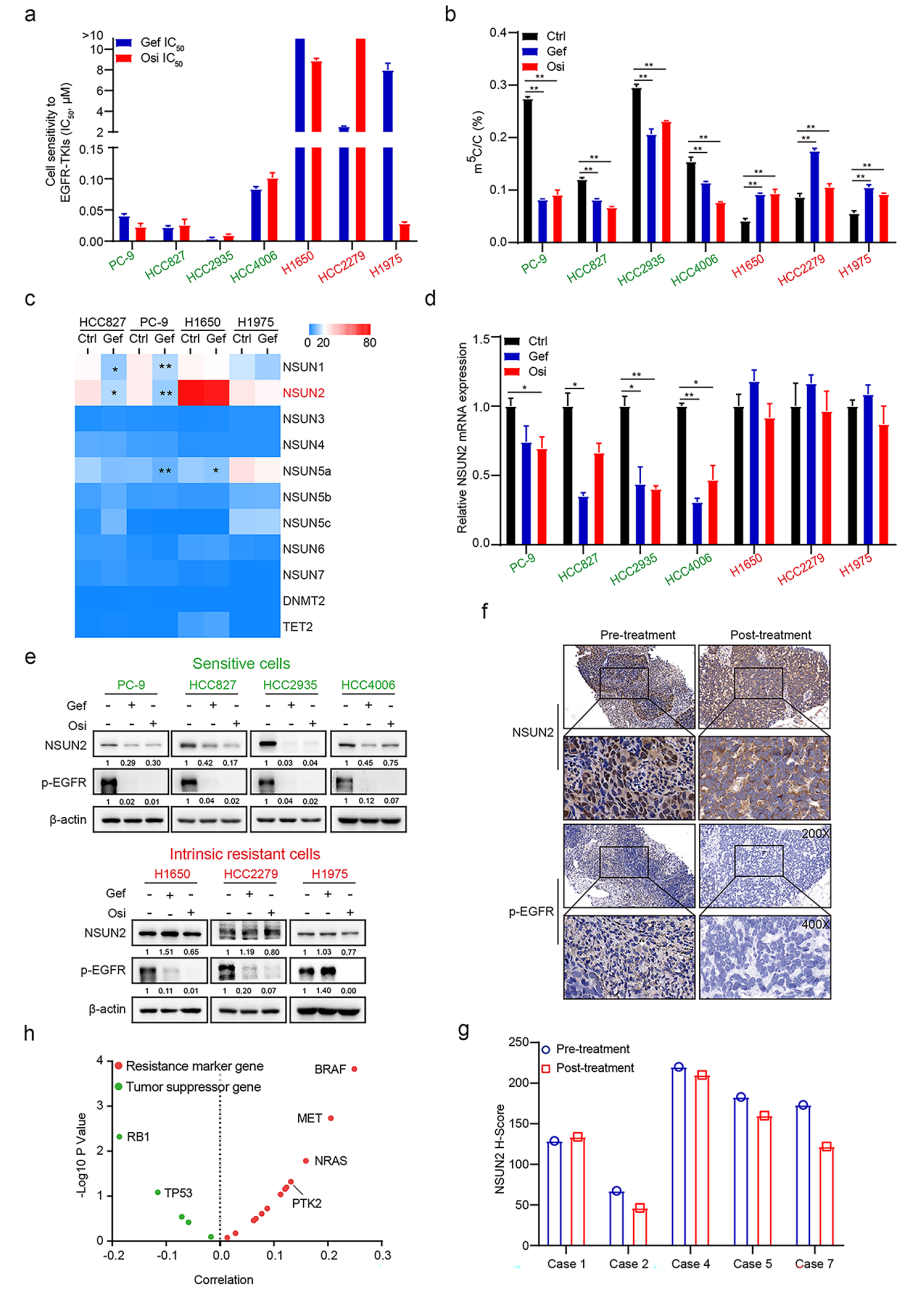
**m**^**5**^**C hypermethylation and stable NSUN2 expression are linked to intrinsic gefitinib resistance in NSCLCs. a** NSCLC cells were treated with gefitinib or osimertinib at gradient concentrations for 72 h and the IC_50_ values were measured using CCK-8 assay. **b** Sensitive and resistant cells were treated with gefitinib (1 µM) or osimertinib (1 µM) for 24 h and the levels of 5-methylcytosine (m^5^C) in purified mRNA were measured by ELISA. **c** Sensitive and resistant cells were treated with gefitinib (1 µM) for 24 h and heatmap for gene expression of RNA m^5^C methyltransferases and demethylase from microarray analysis. Asterisk indicates control *vs* gefitinib group statistically significant difference. **d** Sensitive and resistant cells were treated with gefitinib (1 µM) or osimertinib (1 µM) for 24 h and mRNA expression of NSUN2 was detected by qRT-PCR. **e** Sensitive and resistant cells were treated with gefitinib (1 µM) or osimertinib (1 µM) for 24 h and the whole cell lysates were analyzed by western blotting with indicated antibodies. **f**, **g** Representative images of NSUN2 and p-EGFR IHC staining (**f**) and the quantitative H-scores (**g**) of pre-treatment and post-treatment biopsies of NSCLC patients with intrinsic resistance to gefitinib. Image magnification: 200 × (upper panel) and 400 × (lower panel). **h** Gene expression analysis of correlation between NSUN2 and drug resistance markers or tumor suppressors in TCGA-LUAD dataset. Data are means ± SD of three independent experiments. ns, not significant, *p < 0.05, **p < 0.01. Ctrl, control; Gef, Gefitinib; Osi, Osimertinib.

To elucidate potential mechanisms of intrinsic resistance to EGFR-TKIs, we evaluated response heterogeneity to EGFR-TKIs in seven NSCLC cell lines (PC-9, HCC827, HCC2935, HCC4006, H1650, HCC2279, H1975) by exposing these cells to a series of concentrations of gefitinib or osimertinib. All these cell lines harbor EGFR activating mutations [23]. Interestingly, four cell lines (PC-9, HCC827, HCC2935, HCC4006) were extremely sensitive to EGFR-TKIs, whereas the other three cell lines (H1650, HCC2279, H1975) showed intrinsic resistance to EGFR-TKIs (Fig. 1a). Then we examined RNA m^5^C methylation level in gefitinib-sensitive and intrinsic gefitinib-resistant NSCLC cell lines using ELISA analysis. Unexpectedly, RNA m^5^C methylation levels were substantially down-regulated in sensitive cells but moderately elevated in resistant cells under gefitinib or osimertinib treatment (Fig. 1b). This elevated RNA m^5^C methylation suggested that RNA m^5^C hypermethylation is associated with intrinsic resistance to EGFR-TKIs. Next, we examined specific methyltransferase responsible for RNA m^5^C methylation in NSCLC. RNA-sequencing (RNA-seq) analysis revealed that NSUN2 ranked the highest expression among m^5^C methyltransferase (NSUN1-NSUN7, DNMT2) and demethylase (TET2) in both sensitive and resistant cell lines. Notably, NSUN2 expression was significantly down-regulated in sensitive cells but maintained stable in resistant cells upon EGFR-TKIs treatment (Fig. 1c). Moreover, qRT-PCR and western blotting analysis further confirmed that NSUN2 mRNA and protein levels were significantly down-regulated in sensitive cells, but maintained stable in resistant cells upon EGFR-TKIs treatment (Fig. 1d, e). We also examined NSUN2 expression in gefitinib-sensitive and intrinsic gefitinib-resistant NSCLC patients. Immunohistochemical analysis demonstrated that NSUN2 was slightly increased in intrinsic gefitinib-resistant NSCLC patients compared to gefitinib-sensitive patients (Figure S1a). Furthermore, we found that gefitinib treatment led to lower levels of phosphorylated EGFR, but the levels of NSUN2 expression were maintained in all five NSCLC patients with intrinsic resistance to gefitinib (Fig. 1f, g). Finally, gene expression analysis of TCGA-LUAD dataset validated that NSUN2 expression was positively correlated with gefitinib resistant-associated genes, but negatively correlated with tumor suppressor genes (Fig. 1h). The Kaplan-Meier analysis indicated that both overall survival (OS) rate and relapse free survival (RFS) rate for NSCLC patients with high NSUN2 expression were significantly lower than those for the patients with low NSUN2 expression (Figure S1b, c). These findings suggested that m^5^C hypermethylation and NSUN2 are correlated with intrinsic gefitinib resistance in NSCLC.

### NSUN2 overexpression promotes gefitinib resistance *in vitro* and *in vivo*

**Fig. 2:**
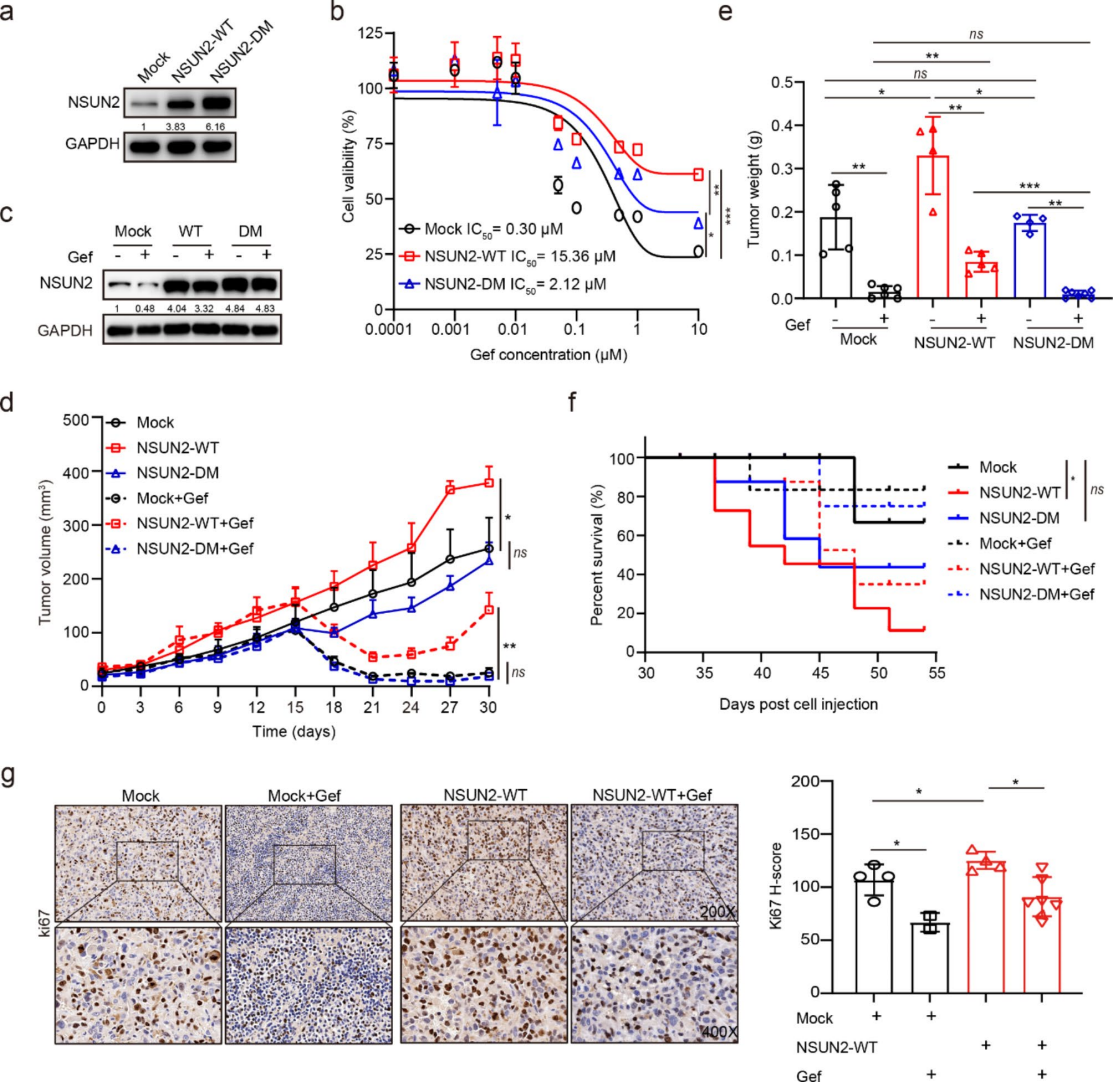
**Overexpression of NSUN2 results in gefitinib resistance and tumor recurrence. a** PC-9 cells stably transfected with wild type NSUN2 (NSUN2-WT), the double catalytic mutant NSUN2 (NSUN2-DM), or empty vector plasmid (Mock) were detected for NSUN2 expression by western blotting analysis. **b** PC-9-Mock, PC-9-NSUN2-WT, PC-9-NSUN2-DM cells were treated with gefitinib at gradient concentrations for 72 h and cell viability as well as gefitinib IC_50_ were measured by CCK-8 assay. **c** PC-9-Mock, PC-9-NSUN2-WT, PC-9-NSUN2-DM cells were treated with gefitinib (1 µM) for 24 h and NSUN2 protein level was determined by western blotting analysis. **d-f** Tumor volume (**d**), tumor weight (**e**), and mice survival (**f**) of xenografts subcutaneously implanted with PC-9-Mock, PC-9-NSUN2-WT, PC-9-NSUN2-DM cells in BALB/c nude mice (n ≥ 6 per group). About two weeks after injection, mice were administrated with 25 mg/kg gefitinib or 0.5% CMC-Na via gavage once daily for consecutive 10 days (from day 15 to day 24). **g** Representative images of ki67 IHC staining and the quantitative H-scores of tumors obtained from tumor xenografts. Image magnification: 200 × (upper panel) and 400 × (lower panel). Data are means ± SD of two independent experiments. For (d), results were expressed as mean ± SEM. For Kaplan-Meier curve, p values were determined using a log-rank test (f). ns, not significant, *p < 0.05, **p < 0.01, ***p < 0.001. Mock, empty vector control; NSUN2-WT, wild type NSUN2; NSUN2-DM, the double catalytic mutant NSUN2.

To determine whether NSUN2-mediated m^5^C hypermethylation contributes to intrinsic resistance to gefitinib, we used lentiviral transduction system to overexpress NSUN2 in gefitinib-sensitive cells for gain-of-function studies (Fig. 2a). Forced expression of wild type NSUN2 (NSUN2-WT) significantly increased cell viability and decreased cell sensitivity to gefitinib treatment in sensitive PC-9 cells compared to the double catalytic mutant NSUN2 (NSUN2-DM, C271A & C321A) (Fig. 2b). Notably, either wild-type or catalytically inactive NSUN2 maintained NSUN2 expression in the presence of gefitinib treatment compared to the control group (Mock), supporting the notion that m^5^C modification mediated by NSUN2 contribute to gefitinib resistance (Fig. 2c). Next, we established xenograft models to evaluate the effect of NSUN2 overexpression on NSCLC progression and gefitinib resistance *in vivo*. As expected, overexpression of NSUN2-WT but not NSUN2-DM led to a rapid tumor formation and increased tumor burden compared to Mock group; meanwhile, overexpression of NSUN2-WT, but not NSUN2-DM, significantly decreased sensitivity to gefitinib treatment compared to the control group; moreover, tumors of NSUN2-WT overexpression were more prone to recurrence after discontinuation of gefitinib treatment, whereas NSUN2-DM had no such effect (Fig. 2d, e). Notably, overexpression of NSUN2-WT but not NSUN2-DM significantly shortened survival of the tumor-bearing mice (p = 0.0233) and decreased efficacy of gefitinib treatment compared to Mock group (Fig. 2f). In line with these results, immunohistochemical analysis indicated that overexpression of NSUN2-WT significantly enhanced ki67 expression in the absence or presence of gefitinib treatment (Fig. 2g). Taken together, these data indicated the important role of m^5^C modification mediated by NSUN2 in gefitinib resistance in NSCLC.

### NSUN2 knockdown overcomes intrinsic gefitinib resistance

**Fig. 3:**
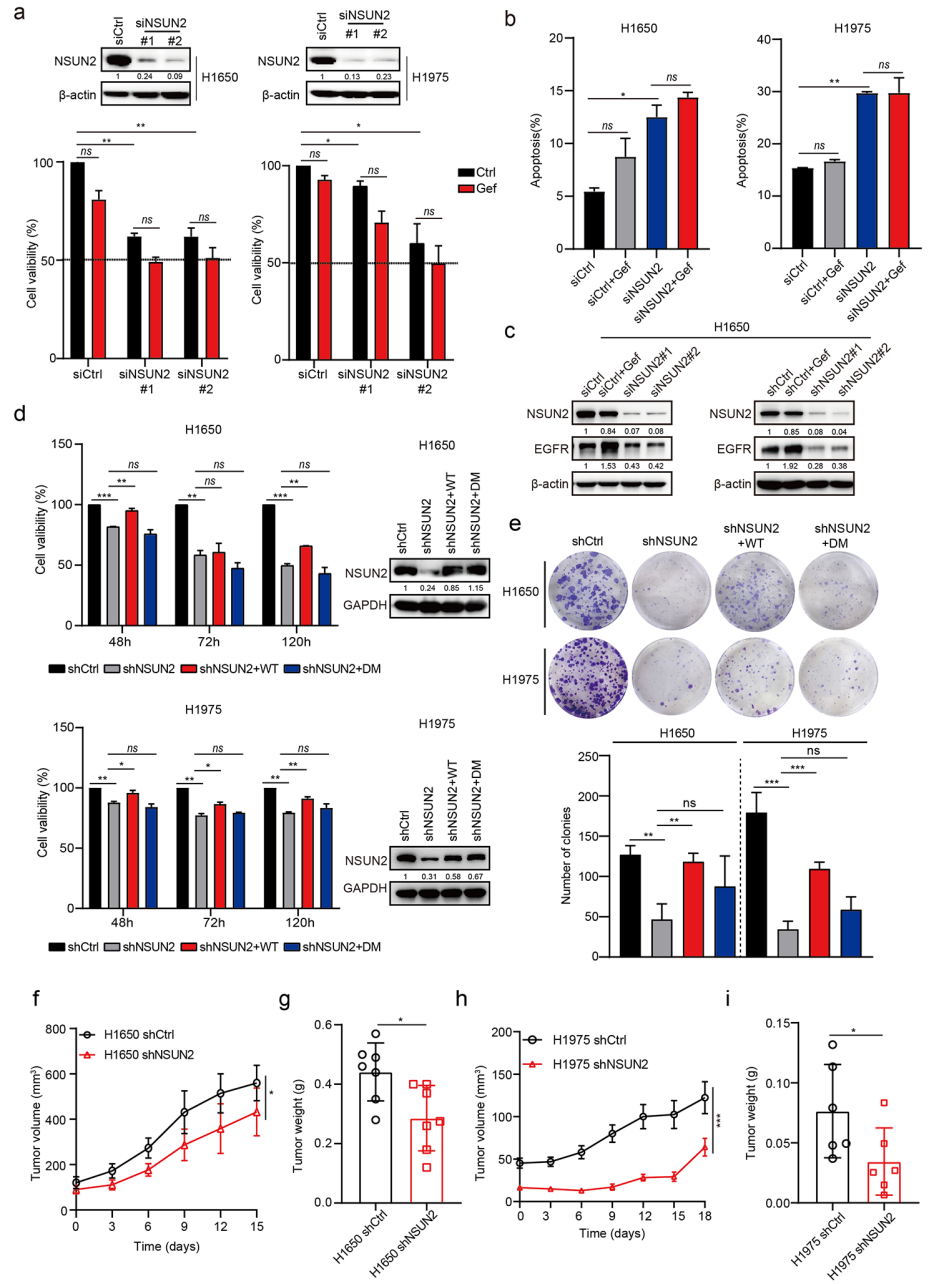
**NSUN2 deficiency overcomes intrinsic gefitinib resistance *in vitro *and *in vivo***. **a** H1650 and H1975 cells transfected with siRNA targeting NSUN2 (siNSUN2) or non-targeting control (siCtrl) were exposed to gefitinib (1 µM) for 72 h and cell viability was measured by CCK-8 assay. NSUN2 knockdown efficacy was evaluated using western blotting (top panel). **b** H1650 and H1975 cells transfected with siNSUN2 or siCtrl were exposed to gefitinib (1 µM) for 72 h and cell apoptosis was detected by Annexin V-FITC/PI staining. **c** H1650 cells transfected with siNSUN2 or shNSUN2 were detected for EGFR protein expression by western blotting analysis. **d, e** H1650 and H1975 cells pre-treated with shNSUN2 were stably transfected with NSUN2-WT or NSUN2-DM and cell proliferation was detected by CCK-8 (**d**) or colony formation assay (**e**). Rescue efficacy of NSUN2 was evaluated by western blotting (right panel). **f, g** Mean volumes (**f**) and tumor weights (**g**) of tumor xenografts obtained from BALB/c nude mice subcutaneously implanted with H1650-shCtrl or H1650-shNSUN2 cells (n = 7 per group). **h, i** Growth curve (**h**) and average tumor weights (**i**) of H1975-shCtrl and H1975-shNSUN2-derived xenografts in the subcutaneous implantation mouse model (n = 6 per group). Error bars represent means ± SD. For (h), results were expressed as mean ± SEM. For a-e, n = 3 biological independent experiments. ns, not significant, *p < 0.05, **p < 0.01, ***p < 0.001.

To determine whether inhibition of NSUN2 could overcome intrinsic resistance to gefitinib, we used small interference RNA (siRNA) treatment to silence NSUN2 expression for loss-of-function studies. Compared with a non-targeting control siRNA (siCtrl), transient depletion of NSUN2 significantly inhibited cell proliferation of intrinsic resistant cells (Fig. 3a) but not sensitive cells (PC-9 and HCC4006) and acquired resistant cells (PC-9AR and PC-9GR) that were endowed with decreased NSUN2 expression upon gefitinib treatment (Figure S2a-d). Meanwhile, we demonstrated that knockdown of NSUN2 induced substantial apoptosis of H1650 and H1975 cells without affecting cell cycle progression (Fig. 3b and Figure S2e). Intriguingly, NSUN2 silencing scarcely increased the sensitivity to gefitinib treatment in resistant cell lines, which may be attributed to the down-regulation of EGFR expression upon NSUN2 knockdown (Fig. 3c). In addition, stable knockdown of NSUN2 by short hairpin RNA (shRNA) also obviously inhibited cell proliferation and decreased the colony formation of intrinsic resistant cells, which could be rescued by stable overexpression of NSUN2-WT but not the catalytic mutant NSUN2-DM (Fig. 3d, e). In line with *in vitro* results, NSUN2 knockdown resulted in a lower rate of tumor formation and reduced tumor burden in subcutaneous xenograft models of gefitinib-resistant H1650 and H1975 cells (Fig. 3f-i). Taken together, our *in vitro* and *in vivo* results indicated that targeting NSUN2 may hold therapeutic potential to overcome intrinsic gefitinib resistance in NSCLC.

### YBX1 promotes gefitinib resistance as an m^5^C reader

**Fig. 4:**
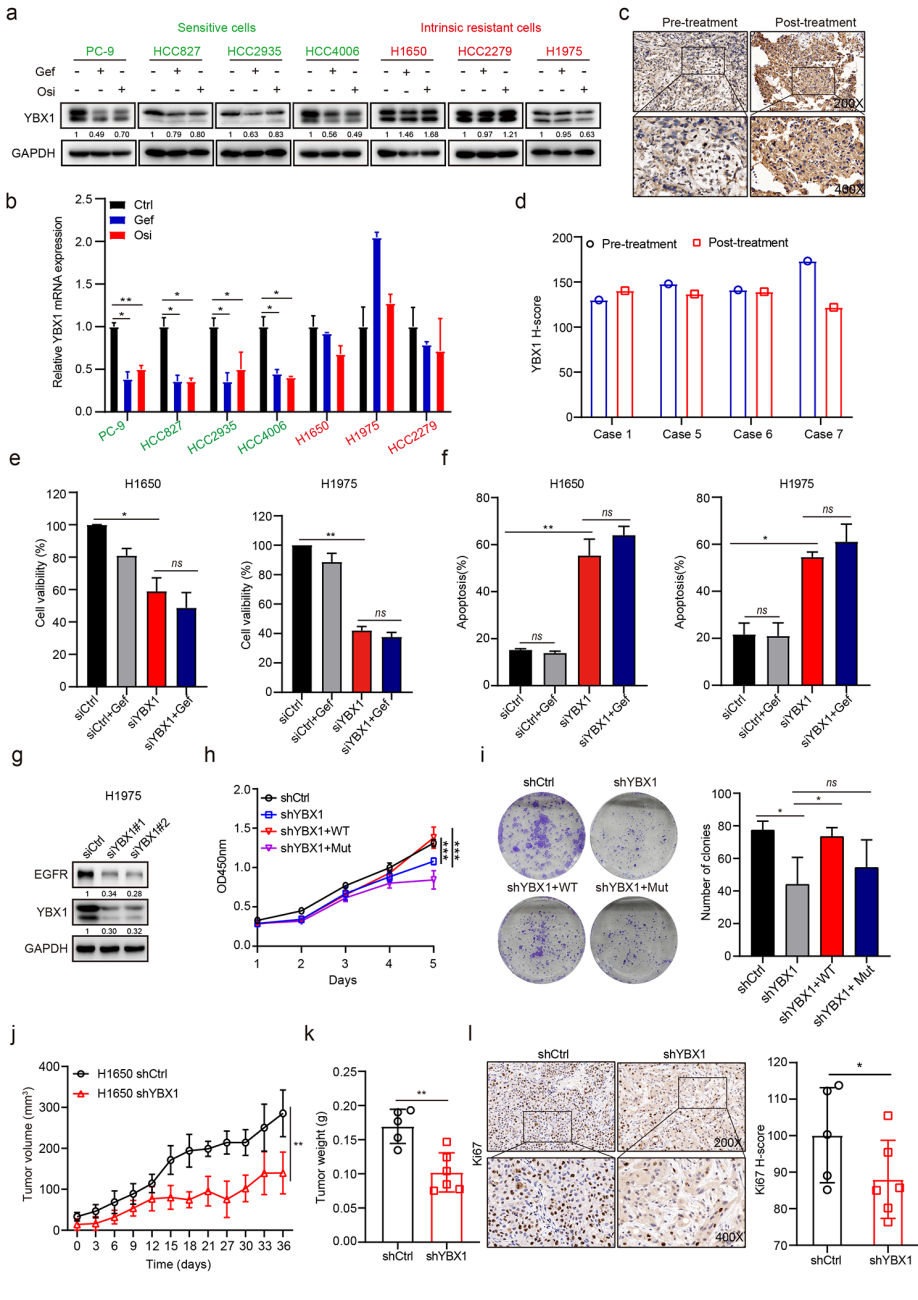
**m**^**5**^**C reader YBX1 expression is required for intrinsic gefitinib resistance. a** Sensitive and resistant cells were treated with gefitinib (1 µM) or osimertinib (1 µM) for 24 h and YBX1 protein level was examined by western blotting. **b** Sensitive and resistant cells were treated with gefitinib (1 µM) or osimertinib (1 µM) for 24 h and YBX1 mRNA expression was determined by qRT-PCR analysis. **c**, **d** Representative images of YBX1 IHC staining (**c**) and the quantitative H-scores (**d**) in pre-treatment and post-treatment biopsies of NSCLC patients with intrinsic resistance to gefitinib. Image magnification: 200 × (upper panel) and 400 × (lower panel). **e, f** H1650 and H1975 cells transfected with siRNA targeting YBX1 (siYBX1) or non-targeting control (siCtrl) were exposed to gefitinib (1 µM) for 72 h and cell viability (**e**) or cell apoptosis (**f**) was respectively detected by CCK-8 assay or Annexin V-FITC/PI staining. **g** H1975 cells transfected with siYBX1 was detected for EGFR protein expression by western blotting analysis. **h, i** H1650 cells pre-treated with shYBX1 were stably transfected with wild type YBX1 (YBX1-WT) or the binding deficient mutant (YBX1-Mut, W65A) and cell proliferation was detected by CCK-8 (**h**) or colony formation assay (**i**). **j**, **k** Tumor growth (**j**) and tumor weights (**k**) of H1650-shCtrl and H1650-shYBX1 cells-derived xenografts subcutaneously implanted in BALB/c nude mice (n ≥ 5). **l** Representative images of ki67 IHC staining and the quantitative H-scores of tumors obtained from H1650-shCtrl and H1650-shYBX1 xenografts. Image magnification: 200 × (upper panel) and 400 × (lower panel). Data are represented as means ± SD. For a, b, e-f, n = 3 biological independent experiments. ns, not significant; *p < 0.05, **p < 0.01, ***p < 0.001.

To gain a more systematic understanding of aberrant m^5^C hypermethylation in intrinsic gefitinib resistance in NSCLC, we then explored the effect of m^5^C reader on intrinsic gefitinib resistance. In both sensitive and resistant NSCLC cells, the expression of YBX1 was significantly higher than that of ALYREF, as revealed by transcriptomic analysis. Moreover, YBX1 levels remained stable under gefitinib treatment in resistant cells compared to the marked downregulation in sensitive cells (Figure S3a). Western blotting and qRT-PCR analysis confirmed that EGFR-TKIs treatment significantly downregulated the expression of YBX1 in sensitive cells but not in resistant cells (Fig. 4a, b). Furthermore, we found that YBX1 expression remained stable in all (4/4) NSCLC patients with intrinsic resistance after gefitinib treatment using immunohistochemistry analysis (Fig. 4c, d). Thus, our data suggested that gefitinib treatment failed to down-regulate m^5^C reader YBX1 in resistant NSCLC cells.

Next, we examined the effects of YBX1 knockdown on intrinsic gefitinib resistance. Depletion of YBX1 by siRNA significantly inhibited cell proliferation and induced remarkable apoptosis of resistant cells (Fig. 4e, f and Figure S3b). Similar to knockdown of NSUN2, knockdown of YBX1 did not increase gefitinib sensitivity due to the down-regulation of EGFR expression upon YBX1 knockdown (Fig. 4g). Meanwhile, lentivirus-mediated YBX1 silencing also obviously inhibited cell proliferation and decreased the colony formation of intrinsic resistant cells, which could be rescued by overexpression of wild type YBX1(YBX1-WT) but not the binding deficient mutant YBX1-Mut (W65A) (Fig. 4h, i). In line with this result, YBX1 knockdown caused a lower rate of tumor formation and reduced tumor burden in gefitinib-resistant subcutaneous xenograft models (Fig. 4j, k). Moreover, knocking down YBX1 led to a significantly lower ki67 expression in the shYBX1 group than the control group (Fig. 4l). Together, these data suggested that YBX1 played a role in gefitinib resistance as an m^5^C reader in NSCLC.

### QSOX1 was identified as a potential target of NSUN2-mediated m^5^C hypermethylation

**Fig. 5:**
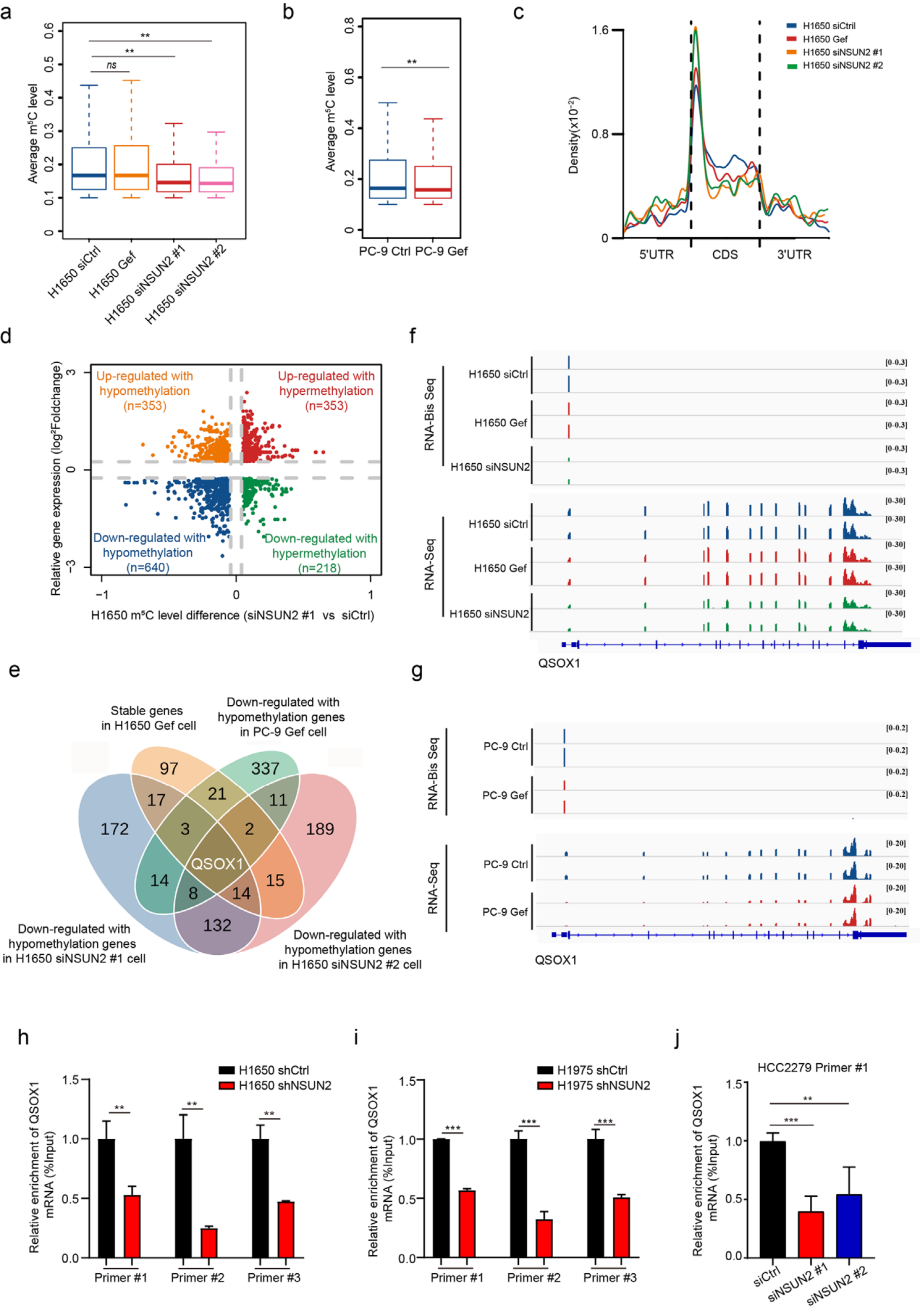
**Epi-transcriptome analysis identifies QSOX1 as an m**^**5**^**C-modified target in resistant NSCLC cells. a, b** H1650 cells (**a**) or PC-9 cells (**b**) were transfected with siNSUN2 for 72 h or treated with gefitinib (1 µM) for 24 h and the average m^5^C level was analyzed via RNA-BisSeq. **c** The m^5^C distributions within different regions in control, NSUN2 knockdown or gefitinib-treated H1650 cells. **d** Significant changes in the m^5^C methylation and mRNA expression levels in H1650 siNSUN2#1 cells. **e** Venn diagram illustrating the potential m^5^C modification candidates of direct NSUN2 targets. **f, g** IGV analysis showed that changes in mRNA expression and m^5^C levels of QSOX1 in H1650 (**f**) and PC-9 (**g**) cells upon NSUN2 knockdown or gefitinib treatment. **h-j** Purified mRNA was immunoprecipitated by anti-m^5^C antibody and the m^5^C levels of QSOX1 in H1650 (**h**), H1975 (**i**), and HCC2279 (**j**) were analyzed by qRT-PCR. Error bars are means ± SD of three independent experiments. ns, not significant, **p < 0.01, ***p < 0.001.

In order to investigate the potential target of NSUN2 involved in intrinsic resistance to EGFR-TKIs in NSCLC, RNA-seq technique combined with RNA bisulfite sequencing (RNA-BisSeq) were performed for whole-transcriptome mapping and profiling. In RNA-seq and RNA-BisSeq data, the Pearson’s correlation and the overlap m^5^C sites were high-confidence between the two biological replicates (siNSUN2#1 and siNSUN2#2) in H1650 cells (Figure S4). On average, the m^5^C methylation level was significantly lower in H1650 cells transfected with siNSUN2 compared to siCtrl (Fig. 5a). Similarly, gefitinib treatment also resulted in lower m^5^C methylation level in PC-9 cells (Fig. 5b). RNA-seq analysis showed that about 54.0% of the transcripts were down-regulated in H1650 cells upon NSUN2 knockdown and generally 58.2% were down-regulated in PC-9 cells with gefitinib treatment (Figure S5a-f). Gene Ontology (GO) analysis revealed that down-regulated genes were significantly enriched in biological processes associated with EGFR-TKIs resistance (Figure S5i). Consistent with previous reports [18], the m^5^C methylation pattern was primarily distributed in the coding sequence (CDS) of the mRNA transcript in NSCLC cells (Fig. 5c and Figure S6a, b). About 63.4% of the m^5^C sites displayed hypomethylation in H1650 siNSUN2 cells (Figure S6c-h). Kyoto Encyclopedia of Genes and Genomes (KEGG) analysis revealed that the hypomethylated m^5^C sites were mainly associated with cancer-related pathways (Figure S6i-k). Additionally, GO analysis revealed that the down-regulated genes with hypomethylated m^5^C in H1650 cells upon NSUN2 knockdown and PC-9 cells with gefitinib treatment were primarily enriched in extracellular matrix organization, extracellular structure organization and mRNA modification (Figure S7a-c).

To identify the target gene, 159 overlapped genes were obtained from significantly down-regulated mRNAs with hypomethylated m^5^C between H1650 siNSUN2#1 and H1650 siNSUN2#2 group (Fig. 5d and Figure S5g, S7d). In addition, we found 401 genes (809 sites) with both down-regulated mRNA and hypomethylated m^5^C in PC-9 cells after gefitinib treatment (Figure S7e). Furthermore, about 174 genes (329 sites) showed stable mRNAs and m^5^C levels in H1650 cells after gefitinib treatment (Figure S7f). Finally, we took the intersection of the above genes and obtained the candidate target gene, QSOX1 (Fig. 5e and Figure S5h). Integrative Genomics Viewer (IGV) analysis showed that the mRNA expression and m^5^C level of QSOX1 significantly decreased in H1650 cells with NSUN2 knockdown and PC-9 cells after gefitinib treatment (Fig. 5f, g). Further, methylated RNA immunoprecipitation (MeRIP) combined with qRT-PCR results indicated that NSUN2 knockdown decreased the m^5^C level of QSOX1 mRNA compared with the control in H1650, H1975 and HCC2279 cells, respectively (Fig. 5h-j). Taken together, these data indicated that QSOX1 was a potential target of NSUN2-mediated intrinsic resistance to gefitinib in NSCLC.

### NSUN2 regulates QSOX1 mRNA translation in an m^5^C-YBX1-dependent manner

Next, we investigated the molecular mechanism by which NSUN2-m^5^C-YBX1 axis regulates QSOX1 expression in promoting intrinsic EGFR-TKIs resistance in NSCLC. qRT-PCR assay demonstrated that NSUN2 deficiency partially decreased mRNA levels of QSOX1 in resistant cells (Figure S8a). Similarly, YBX1 knockdown partially reduced mRNA expression of QSOX1 in resistant cells (Figure S8b). To further investigate whether NSUN2/YBX1 mediates the stabilization of QSOX1, we used RNA synthesis inhibitor actinomycin-D to find that NSUN2/YBX1 knockdown barely decreased the half-life of QSOX1 mRNA (Figure S8c).

**Fig. 6:**
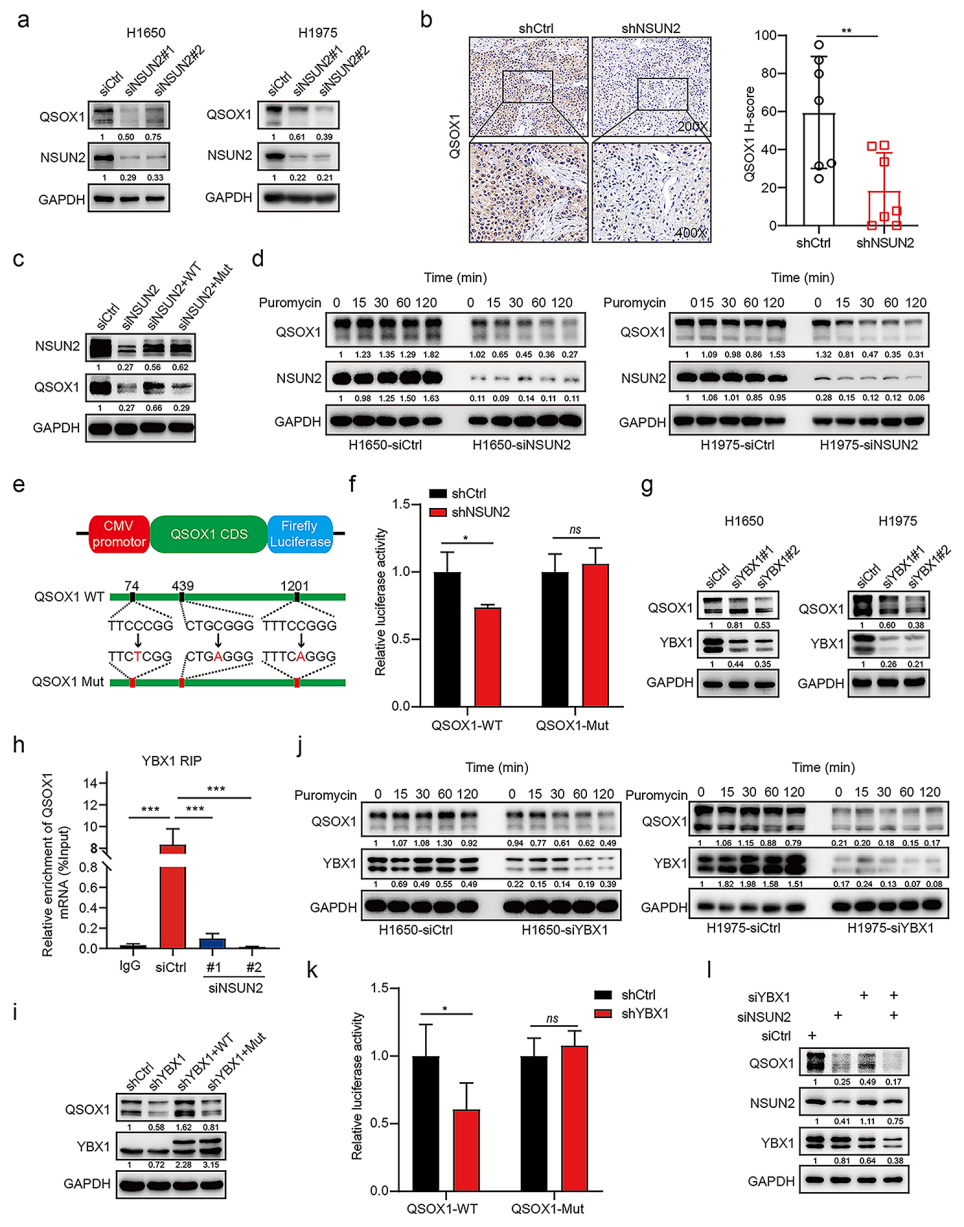
**NSUN2 and YBX1 regulate QSOX1 expression via manipulation of mRNA translation. a** H1650 and H1975 cells were transfected with siNSUN2 or siCtrl for 72 h and the whole cell lysates were detected by western blotting with indicated antibodies. **b** Representative images of QSOX1 IHC staining and the quantitative H-scores of tumors obtained from the H1650-shCtrl and H1650-shNSUN2 xenografts. Image magnification: 200 × (upper panel) and 400 × (lower panel). **c** H1650 cells pre-treated with siNSUN2 were transfected with pcDNA-NSUN2-WT or pcDNA-NSUN2-Mut (C271A&C321A) plasmid and western blotting analysis of QSOX1 expression. **d** H1650 or H1975 cells transfected with siCtrl or siNSUN2 were treated with 200 nM puromycin for the indicated time and the whole cell lysates was detected by western blotting with indicated antibodies. **e** QSOX1 CDS containing either wild type or mutant (C-to-T/A mutation) m^5^C sites was cloned into luciferase reporter vector. **f** Relative luciferase activity of the wild-type and mutant form of QSOX1 CDS reporter vectors in H1650 cells transfected with shCtrl or shNSUN2, respectively. **g** The expression of QSOX1 in H1650 or H1975 cells transfected with siCtrl or siYBX1 was determined by western blotting analysis. **h** RNA immunoprecipitation of YBX1 and QSOX1 mRNA was carried out in H1650 cells transfected with siCtrl or siNSUN2 using anti-YBX1 antibody, with IgG as the control. **i** H1650 cells pre-treated with shYBX1 were transfected with 3FLAG-YBX1-WT or 3FLAG-YBX1-Mut (W65A) plasmid and western blotting analysis of QSOX1 expression. **j** H1650 or H1975 cells transfected with siCtrl or siYBX1 were treated with 200 nM puromycin for the indicated time and the whole cell lysates were detected by western blotting with indicated antibodies. **k** Relative luciferase activity of the wild-type and mutant form of QSOX1 CDS reporter vectors in H1650 cells transfected with shCtrl or shYBX1, respectively. **l** H1650 cells were transfected with siNSUN2 or siYBX1 alone or in combination for 72 h and the whole cell lysates was detected by western blotting with indicated antibodies. Error bars are means ± SD of three independent experiments. ns, not significant; *p < 0.05, **p < 0.01, ***p < 0.001.

On the basis of these results, we next determined the protein expression of QSOX1 following NSUN2 inhibition. Two short interfering RNA fragments of NSUN2 consistently led to obviously reduced QSOX1 protein abundance in resistant cells (Fig. 6a). In addition, immunohistochemical analysis validated that NSUN2 knockdown significantly reduced QSOX1 protein level in H1650 xenograft tumors (Fig. 6b). Rescue with wild type NSUN2, but not the catalytic mutant NSUN2 (NSUN2-Mut), restored the reduced QSOX1 protein level caused by NSUN2 knockdown in intrinsic resistant cells (Fig. 6c), suggesting a role of m^5^C-modification mediated by NSUN2 in regulating QSOX1 expression level. Furthermore, NSUN2 knockdown markedly inhibited QSOX1 global mRNA translation compared to control cells reflected by puromycin incorporation assay (Fig. 6d and Figure S8d). Based on the bioinformatic analysis that NSUN2-regulated m^5^C modification regions are mainly at the CDS of QSOX1 mRNA, we constructed wild-type (QSOX1-WT) and mutant QSOX1 CDS (QSOX1-Mut) luciferase reporter plasmids (Fig. 6e). As expected, knockdown of NSUN2 significantly decreased luciferase activity in the QSOX1-WT group, not in the QSOX1-Mut group (Fig. 6f). These results indicated that NSUN2 might regulate QSOX1 protein levels in an m^5^C-dependent manner.

In addition, YBX1 knockdown markedly decreased protein expression levels of QSOX1 in resistant cells (Fig. 6g). RNA immunoprecipitation (RIP)-qPCR assay showed that less YBX1 was bound to QSOX1 mRNA in the NSUN2 depletion group compared to the control group (Fig. 6h), which indicates that YBX1-mediated QSOX1 regulation is m^5^C-dependent. Rescue with wild-type YBX1, but not the binding deficient mutant YBX1, restored the protein level of QSOX1 in YBX1-knockdown intrinsic resistant cells (Fig. 6i). Moreover, protein synthesis assay showed that stable YBX1 knockdown completely inhibited mRNA translation activity (Fig. 6j and Figure S8e). In support of these, knockdown of YBX1 significantly decreased luciferase activity in the QSOX1-WT reporter plasmid; however, knockdown of YBX1 showed no effect on luciferase activity in the mutated QSOX1 reporter plasmid (Fig. 6k). Importantly, simultaneous knockdown of NSUN2 and YBX1 did not further reduce QSOX1 expression compared with NSUN2 knockdown or YBX1 knockdown alone, suggesting that NSUN2 regulated QSOX1 protein levels in an m^5^C-YBX1-dependent manner (Fig. 6l). Collectively, these data indicated that NSUN2 regulates QSOX1 mRNA translation in an m^5^C-YBX1-dependent manner in resistant NSCLC cells.

### QSOX1 is associated with intrinsic gefitinib resistance

**Fig. 7:**
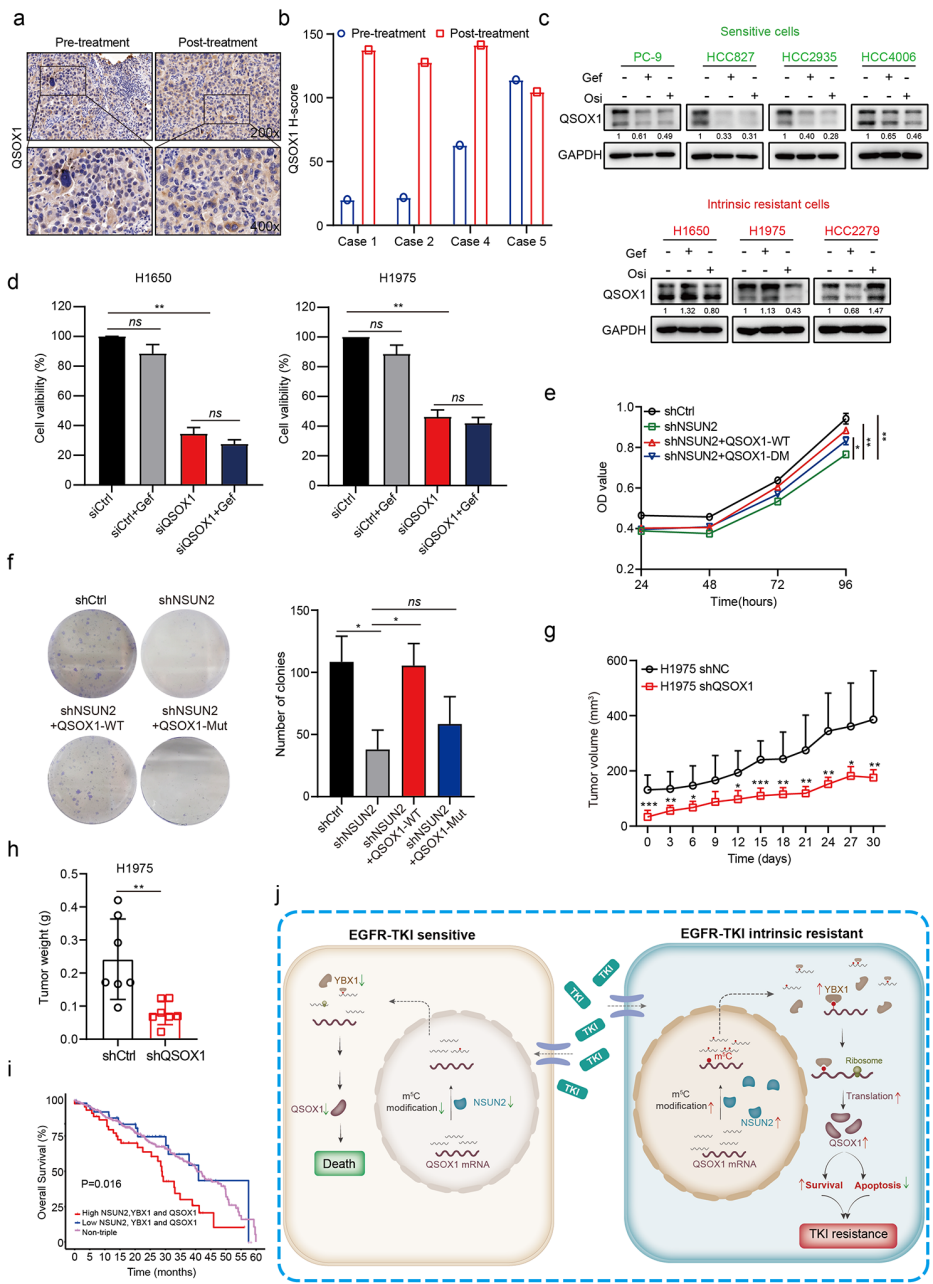
**The abundance of QSOX1 controls sensitivity of NSCLCs to gefitinib**. **a**, **b** Representative images of QSOX1 IHC staining (**a**) and the quantitative H-scores (**b**) in pre-treatment and post-treatment biopsies of NSCLC patients with intrinsic resistance to gefitinib. Image magnification: 200 × (upper panel) and 400 × (lower panel). **c** Sensitive and resistant cells were treated with gefitinib (1 µM) or osimertinib (1 µM) for 24 h and QSOX1 protein level was detected by western blotting. **d** H1650 and H1975 cells transfected with siRNA targeting QSOX1 (siQSOX1) or non-targeting control (siCtrl) were exposed to gefitinib (1 µM) for 72 h and cell viability was detected by CCK-8 assay. **e**, **f** H1650 cells pre-treated with shNSUN2 were stably transfected with wild type QSOX1 (QSOX1-WT) or QSOX1 with the mutated m^5^C sites (QSOX1-Mut) and cell proliferation was detected by CCK-8 (**e**) or colony formation assay (**f**). **g, h** Tumor growth curve (**g**) and tumor weights (**h**) of xenografts subcutaneously implanted with H1975-shCtrl and H1975-shQSOX1 cells in BALB/c nude mice (n = 7 per group). **i** Kaplan-Meier analysis of TCGA-LUAD dataset showing the combined high expression of NSUN2, YBX1 and QSOX1 predicting a poorer overall survival. P value (p = 0.016) was determined using a log-rank test. **j** Working model for aberrant m^5^C hypermethylation conferring intrinsic resistance to gefitinib in NSCLC via NSUN2/YBX1/QSOX1 axis. The results are shown as means ± SD. For c-e, n = 3 biological independent experiments. *p < 0.05, **p < 0.01, ***p < 0.001.

We further investigated the role of QSOX1 in intrinsic gefitinib resistance in NSCLC. Immunohistochemical analysis demonstrated that the expression of QSOX1 was slightly but not significantly increased in gefitinib-resistant NSCLC tissues compared to gefitinib-sensitive patient samples (Figure S9a). Moreover, QSOX1 expression was increased or maintained stable in all (4/4) intrinsic resistant patients after gefitinib treatment (Fig. 7a, b). qRT-PCR and western blotting analysis confirmed that QSOX1 mRNA and protein expression were significantly decreased in sensitive cells upon gefitinib or osimertinib treatment, but maintained stable in resistant cells (Fig. 7c and Figure S9b). Thus, these data suggested that gefitinib failed to down-regulate QSOX1 expression in intrinsic gefitinib-resistant NSCLC cells.

Next, we examined the effects of QSOX1 knockdown on intrinsic gefitinib resistance. Transient knockdown of QSOX1 significantly inhibited cell viability of intrinsic resistant cells (Fig. 7d and Figure S9c). Notably, wild type QSOX1 (QSOX1-WT) expression largely rescued defective cell proliferation and colony formation in NSUN2 knockdown cells compared with QSOX1 with the mutated m^5^C sites (QSOX1-Mut), indicating QSOX1 is a functional target of NSUN2 in intrinsic gefitinib resistance (Fig. 7e, f). Additionally, QSOX1 knockdown promoted cell apoptosis and increased PARP and Caspase-3 cleavage in resistant cells (Figure S9d, e). Furthermore, immunofluorescence analysis confirmed that QSOX1 knockdown, alone or in combination with gefitinib treatment, led to the accumulation of cleavage of Caspase-3 protein (Figure S9f). In line with *in vitro* results, QSOX1 knockdown caused a lower rate of tumor formation and reduced tumor burden in gefitinib-resistant subcutaneous xenograft models (Fig. 7g, h). Finally, analysis of TCGA database revealed that the combined high expression of NSUN2, YBX1 and QSOX1 predicted the poorest overall survival in lung adenocarcinoma (LUAD) (Fig. 7i), breast cancer (BRCA) and hepatocellular carcinoma (HCC) (Figure S9g). Together, these results suggest that QSOX1 is associated with intrinsic gefitinib resistance in NSCLC cells and the NSUN2-YBX1-QSOX1 axis plays crucial roles in intrinsic resistance to EGFR-TKIs in NSCLC.

## Discussion

EGFR-targeted therapy is the standard first-line treatment for advanced NSCLC patients with activating EGFR mutations [24], but approximately 30% of patients demonstrate intrinsic resistance to EGFR-TKIs [25]. Emerging evidence revealed that METTL3-induced m^6^A modification played an important role in acquired resistance to tyrosine kinase inhibitors in leukemia cells [26], PLX4032 resistance in melanoma [27], and cisplatin resistance in epithelial ovarian cancer [28]. However, the molecular mechanisms of RNA methylation (such as m^5^C and m^6^A) in intrinsic resistance to EGFR-TKIs remain largely unknown.

We here find differential m^5^C methylation abundance between sensitive and intrinsic resistant NSCLC cells after EGFR-TKIs treatment. In contrast, our data show no detectable difference of m^5^C methylation between sensitive and intrinsic resistant NSCLC cells before EGFR-TKIs treatment. This may be attributed to the fact that intrinsic resistance is not detected until after targeted therapy [25]. The universally elevated m^5^C methylation in intrinsic EGFR-TKIs-resistant cells mainly results from the maintained NSUN2 expression after EGFR-TKIs treatment. Consistent with this, we see a substantial decrease in m^5^C abundance and NSUN2 expression in EGFR-TKIs-sensitive NSCLC cells. Consequently, overexpression of wild type NSUN2, but not the catalytic mutant NSUN2, leads to gefitinib resistance and tumor recurrence, while depletion of NSUN2 results in tumor regression and overcomes intrinsic resistance to gefitinib. Interestingly, the correlation between NSUN2-mediated m^5^C hypermethylation and gefitinib resistance exists only in the intrinsic resistant cells, but not in the acquired resistant cells that have lower NSUN2 levels upon gefitinib treatment. This indicates that m^5^C hypermethylation, unlike m^6^A methylation, may not be involved in acquired TKIs resistance [26]. Our results support the previous notion that the molecular mechanism of intrinsic resistance to EGFR-TKIs is different from that of acquired resistance in NSCLC [29].

FTO expression was suppressed by R-2HG treatment in sensitive leukemia cells through downregulation of CEBPA transcription factor but not in resistant cells [30]. Similarly, we find that gefitinib treatment decreases NSUN2 expression in sensitive cells but increases or maintains these NSUN2 expression in intrinsic resistant cells *in vitro* and *in vivo* (data not shown). The mechanism underlying the negative correlation between NSUN2 expression level and gefitinib sensitivity in EGFR mutant NSCLC cells are under investigation. The combination of NSUN2 depletion and gefitinib treatment has no significantly additive inhibitory effect in intrinsic resistant cells, likely due to the decreased EGFR expression after NSUN2 depletion. This observation is different from a recent publication which reported that the combination of FTO depletion and BCR-ABL inhibitors showed significantly additive inhibitory effect in acquired resistant leukemia cells [31].

QSOX1 can insert disulfide bonds into unfolded proteins with the concomitant reduction of oxygen to hydrogen peroxide [32, 33]. QSOX1 expression is upregulated in quiescent fibroblast cells and plays a role in the formation of extracellular matrix [34]. Previous studies showed that QSOX1 promoted sorafenib-induced ferroptosis in hepatocellular carcinoma and could be a useful lung cancer biomarker as well as a potential therapeutic target for lung cancer [35–37]. However, the function and molecular mechanism underlying QSOX1 upregulation in drug resistance remains to be identified. Here, we identify QSOX1 as a potential target of NSUN2 and YBX1 in an m^5^C-modification-dependent mechanism in intrinsic gefitinib resistant NSCLC. NSUN2 catalyzes the formation of m^5^C at the CDS region of QSOX1 mRNA and YBX1 recognizes m^5^C-containing QSOX1 mRNA. It has been reported that NSUN2-YBX1-mediated m^5^C modification maintained the stability of HDGF mRNA in human urothelial carcinoma of the bladder [18]. In this study, we find that the effect of NSUN2-YBX1-mediated m^5^C modification on QSOX1 mRNA stability is slight, but the effect on QSOX1 mRNA translation is comprehensive and profound. Previous studies reported that YBX1 affected mRNA translation of HIF1α and MYC to promote sarcoma metastasis and leukemia progression at a posttranscriptional level [38–40]. Further study of the detailed mechanism of the QSOX1 mRNA translation regulated by the NSUN2-YBX1 signaling is warranted.

## Conclusions

In summary, the present study reveals that the NSUN2-YBX1-QSOX1 axis is a previously unappreciated mechanism that regulates intrinsic resistance to EGFR-TKIs in NSCLC (Fig. 7j). Inhibition of the NSUN2-YBX1-QSOX1 axis overcomes intrinsic gefitinib resistance in m^5^C-dependent regulatory mechanism. Our findings highlight the NSUN2-YBX1-QSOX1 axis as candidate biomarkers for the prognosis and treatment of NSCLC patients with intrinsic resistance to EGFR-TKIs.

## Materials and methods

**Cell lines and reagents**. The human lung adenocarcinoma cell lines HCC827, HCC4006, HCC2935, H1975, HCC2279 and H1650 cells were obtained from American Type Culture Collection (ATCC). PC-9 was kindly gifted by Prof. Jian Ding (Shanghai Institute of Materia Medica, Shanghai, China). All the cell lines were cultured at 37 °C in a humidified 5% CO_2_ incubator in RPMI-1640 medium supplemented with 10% fetal bovine serum (FBS) (Gibco). Gefitinib and osimertinib were purchased from Selleck Chemicals and dissolved to 10 mmol/L with DMSO as stock solutions for *in vitro* studies.

**Patient samples.** The pathological biopsies of lung adenocarcinoma tissues were collected from NSCLC patients with drug sensitivity or intrinsic resistance to EGFR-TKIs in the First Affiliated Hospital of Zhengzhou University (Zhengzhou, China). Patients that received surgery, chemoradiotherapy or immunotherapy within the past six months were excluded. All tumor samples had either an exon 19 deletion (19del) or an exon 21 point mutation (L858R) in EGFR, but lacked the EGFR T790M mutation. Intrinsic resistance to EGFR-TKIs was defined as stable disease (SD) < 3 months or progression on the first imaging evaluation after EGFR-TKIs treatment. Clinical specimens of only intrinsic-resistant patients were taken before and after EGFR-TKIs treatment, because re-biopsy is important for exploring resistance mechanisms to select new therapeutic drugs for intrinsic-resistant patients. This study was approved by the Institutional Review Board of the First Affiliated Hospital of Zhengzhou University (the approval number 2019-KY-31) and complied with the Declaration of Helsinki. All subjects provided the written informed consent according to the institutional guidelines.

**Cell viability assay.** Cells were seeded at a density of 5 × 10^3^ cells/well in 96-well plates in RPMI-1640 medium containing 10% FBS. After 24 h, cells were exposed to the indicated concentrations of gefitinib for another 24 h. Then 10 µL of CCK-8 reagent (Vazyme, China) was added to each well for 1 h at 37 °C, and the absorbance (optical density, OD) was read at a wavelength of 450 nm using EnVision® multimode plate reader (PerkinElemer). The IC_50_ values were calculated by concentration-response curve fitting using the four-parameter method.

**Colony formation assay.** Cells were seeded into 6-well plates at a density of 500–1000 cells per well. The medium was exchanged every 3 days for 3 weeks. Colonies were treated with fixation solution (10% methanol + 10% acetic acid) at room temperature for 15 min and then stained with a solution of 1% crystal violet in methanol for 15 min.

**Flow cytometry detection.** Cells were seeded in 6-well plates at a density of 2.5 × 10^5^ cells/well. After 24 h, the cells were transfected with indicated siRNA and treated with DMSO or gefitinib (1 µM) for 72 h. For the cell cycle analysis, cells were fixed with 75% ethanol and then stained with PI/RNase (Beyotine, China) for 15 min at 37 °C. For the cell apoptosis analysis, the cells were stained with annexin V-FITC and PI according to Annexin V-FITC/PI Apoptosis Detection Kit (Vazyme, China). Then, samples were analyzed using a FACS Calibur flow cytometer (BD Biosciences).

**Quantitative real-time PCR (qRT-PCR).** The total RNA was extracted using TRIzol reagent (Invitrogen) and reversely transcribed to complementary DNA (cDNA) using iScript cDNA Synthesis Kit (Bio-Rad). Real-time PCR was performed using iTaq Univer SYBR Green Supermix (Bio-Rad) according to the manufacturer’s instructions. GAPDH or 18 S rRNA was used as a housekeeping gene for normalization. Results were represented as fold expression. The primer pairs used for qPCR analysis were listed below:

NSUN2 forward: 5’- CAAGCTGTTCGAGCACTACTAC-3’,

NSUN2 reverse: 5’- CTCCCTGAGAGCGTCCATGA-3’;

YBX1 forward: 5’- GCGGGGACAAGAAGGTCATC-3’;

YBX1 reverse: 5’- CGAAGGTACTTCCTGGGGTTA-3’;

QSOX1 forward: 5’- TGAGAAAGTTTGGTGTCACCG-3’;

QSOX1 reverse: 5’- GGACCTGGATTCCATGAGCAC-3’;

GAPDH forward: 5’- GGAGCGAGATCCCTCCAAAAT-3’;

GAPDH reverse: 5’- GGCTGTTGTCATACTTCTCATGG-3’;

18S rRNA forward: 5’- GTAACCCGTTGAACCCCATT-3’;

18S rRNA reverse: 5’- CCATCCAATCGGTAGTAGCG-3’.

**Western blotting analysis**. Total protein was extracted using RIPA lysis buffer (Beyotime, China) supplemented with protease inhibitors (Roche) and phosphatase inhibitor cocktail (Sigma) and protein concentration was determined using BCA Protein Assay Kit (Beyotime, China). Harvested lysates were resolved by SDS-PAGE, transferred to nitrocellulose membranes, probed with primary antibodies and then incubated with horseradish peroxidase-conjugated secondary antibodies. The immunoreactive bands were visualized using the chemiluminescence (Thermo Scientific). Primary antibodies used in this study were listed as follows: anti-NSUN2 (Sigma, HPA037896), anti-EGFR (Cell Signaling Technology, #4267), anti-p-EGFR (Cell Signaling Technology, #3777), anti-QSOX1 (Abcam, ab235444), anti-YBX1 (Abcam, ab76149), anti-Cleaved PARP (Cell Signaling Technology, #5625), anti-Cleaved Caspase 3 (Cell Signaling Technology, #9661), anti-β-actin (Cloud Clone, CAB340Mi01) and anti-GAPDH (Cloud Clone, CAB932Mi01).

**Immunohistochemistry (IHC) staining.** Briefly, paraffin sections were heated at 60 °C for 4 h and deparaffinized with BioDewax and Clear Solution (ServiceBio, China). Heat-induced antigen retrieval was carried out using citric acid (PH 6.0) antigen retrieval buffer (ServiceBio, China) for 23 min at 95 °C. Endogenous peroxidase activity of tissues was blocked with 3% hydrogen peroxide at room temperature in darkness for 25 min. Then slides were blocked with 3% BSA for 30 min at room temperature and incubated with primary antibody overnight at 4 °C. Afterward, slides were incubated with horseradish peroxidase conjugated secondary antibodies at room temperature for 50 min. Images were scanned using the 3DHISTECH PANNORAMIC VIEWER. The concentration of anti-p-EGFR (Cell Signaling Technology, #3777), anti-NSUN2 (Sigma, HPA037896), anti-YBX1 (Abcam, ab76149), anti-QSOX1 (Abcam, ab235444), and ki67 (Servicebio, GB111499) were used according to supplier’s instructions.

**Immunofluorescence (IF) staining.** Briefly, cell climbing slides were permeabilization with permeabilize working solution (ServiceBio, China) for 20 min. Then slides were blocked with 3% BSA for 30 min at room temperature and incubated with primary antibody (anti-Cleaved Caspase 3, Cell Signaling Technology, #9661, 1:300 dilution) at 4 °C overnight. Afterward, the slides were incubated with secondary antibody (Cy3 goat anti-rabbit IgG (H + L), Abclonal, AS007, 1:200 dilution) for 1 h at room temperature in 3% BSA/PBS. Nuclei were stained with DAPI (Servicebio, China), and the slides were observed and imaged under a fluorescent microscope (ZEISS).

**RNA interference.** Cells were seeded in 6-well or 96-well plates at 40% confluence. After 24 h, cells were transfected with the indicated siRNA oligonucleotides using Lipofectamine RNAiMAX (Invitrogen) according to the manufacturer’s instructions. Then, the cells were cultured for 72 h and harvested either for cell viability assay or western blotting analysis. The target sequences of siRNA oligonucleotides were as follows:

siNSUN2#1: 5’- GAGAUCCUCUUCUAUGAUCTT-3’;

siNSUN2#2: 5’- CACGUGUUCACUAAACCCUAUTT-3’;

siYBX1#1: 5’- GGAUAUGGUUUCAUCAACATT-3’;

siYBX1#2: 5’- CGUAACCAUUAUAGACGCUTT-3’;

siQSOX1: 5’- CCGGACAATGAAGAAGCCTTT-3’.

**Plasmids and transfection.** pcDNA3.1-NSUN2-WT, pcDNA3.1-NSUN2-Mut (C271/321A), PCMV-YBX1-WT, PCMV-YBX1-Mut (W65A), PLKO.1-shNSUN2 and PLKO.1-shYBX1 plasmids were kindly provided by Prof. Yun-Gui Yang (Beijing Institute of Genomics, Beijing, China). The plasmids GV493-shQSOX1, 3FLAG-EGFP-NSUN2-WT, 3 FLAG-EGFP-NSUN2-DM (C271/321A), 3FLAG-EGFP-YBX1-WT, 3FLAG-EGFP-YBX1-Mut (W65A), 3FLAG-EGFP-QSOX1-WT and 3FLAG-EGFP-QSOX1-Mut (C439/1201A) were purchased from GENECHEM (Shanghai, China). Transient transduction was performed using Lipofectamine 3000 (Invitrogen) according to the manufacturer’s instructions. Then, the cells were cultured for 48 h and harvested for western blotting analysis. For lentiviral transduction, a second-generation lentivirus packaging system consisting of psPAX2 (Addgene) and PMD2.G (Addgene) was used to create virus particles. In brief, the plasmids were transfected into HEK293T packaging cells at 60% confluence using Lipofectamine 3000 (Invitrogen) according to the manufacturer’s instructions. After an additional 48 h incubation, the supernatant was collected, filtered using a 0.45-µm filter (Millipore), and used to infect host cells in the presence of 6 µg/mL polybrene (Solarbio, China). The resultant stable polyclonal populations of transduced cells were then selected with puromycin or hygromycin (Solarbio, China) for two weeks, followed by validation by western blotting.

The shRNA sequences are:

shNSUN2#1: 5’- GCTGGCACAGGAGGGAATATA-3’;

shNSUN2#2: 5’- CACGTGTTCACTAAACCCTAT-3’;

shYBX1: 5’- GGTTCCCACCTTACTACAT-3’;

shQSOX1: 5’- CCGGACAATGAAGAAGCCTTT-3’.

**RNA stability assay.** Cells were seeded in 6-well plates, and then treated with Actinomycin D (5 µg/mL, MedChemExpress) for 0, 2, 4, 6, and 8 h. The same number of H1650 cells were collected and total RNA was extracted by TRIzol reagent (Invitrogen). An equal-volume RNA was reverse-transcribed into cDNA, and then qRT-PCR was used to detect the mRNA level of QSOX1. The relative abundance of mRNA at each time point relative to t = 0 time point was calculated.

**Protein translation assay.** Protein translation levels were determined *in vitro* utilizing the puromycin (P8230, Solarbio, China). Cells were treated with 200 ng/mL puromycin for indicated time points. The cells were lysed, and the protein expression was assayed by western blotting with GAPDH as the reference.

**Dual-luciferase reporter assay.** Luciferase reporter plasmids containing CDS-QSOX1-wild type or CDS-QSOX1-mutant (C439/1201A/C74T) were synthesized by Genechem (Shanghai, China) and cloned into GV272 vector. NSUN2 knockdown cells were plated in a 96-well plate and transfected with 200 ng luciferase reporter plasmids and 10 ng of Renilla luciferase control reporter vectors using Lipofectamine 3000. Then cells were lysed after 48 h and subjected to the luciferase activity analysis using a Dual Luciferase Reporter Assay Kit (E1910, Promega). The firefly-luciferase activity was measured using the EnVision® multimode plate reader (PerkinElemer) and normalized by the renilla-luciferase activity.

**RNA immunoprecipitation (RIP).** H1650 cells were harvested and washed twice with cold PBS, and the cell pellet was incubated with RIP lysis buffer (150 mM KCl, 10 mM HEPES pH 7.6, 2 mM EDTA, 0.5% NP-40, 0.5 mM DTT, Protease Inhibitor, RNase Inhibitor) on ice for 30 min. One tenth portion of the cell lysate was used as input. The rest of the cell lysate was incubated with either Rabbit IgG-coated beads or anti-YBX1 (Abcam, ab76149)-coated beads for 4 h at room temperature. Afterward, the beads-antibody-protein-RNA complex was washed five times with ice-cold washing buffer (200 mM NaCl, 50 mM HEPES pH 7.6, 2 mM EDTA, 0.05% NP-40, 0.5 mM DTT, RNase inhibitor). Then, immunoprecipitated sample was digested with proteinase K and the RNA was precipitated with glycogen (Thermo Scientific, AM9516).

. Total RNA was extracted by TRIzol reagent followed by quantitative RT-PCR.

**Animal studies.** The nu/nu athymic BALB/c female mice (6–8 weeks old) were purchased from Beijing Vital River Laboratory Animal Technology Co., Ltd. (Beijing, China) and maintained under specific pathogen-free conditions. For xenograft implantation, PC-9-Mock, PC-9-NSUN2-WT, PC-9-NSUN2-DM, H1650-shCtrl, H1650-shNSUN2, H1650-shYBX1, H1975-shCtrl, H1975-shQSOX1 cells (6.0 × 10^6^ cells/100 µL) were suspended in PBS and subcutaneously injected into the right flank of all mice. About two weeks after injection with PC-9-Mock, PC-9-NSUN2-WT, PC-9-NSUN2-DM cells, mice were administrated with 25 mg/kg gefitinib (MB1112, meilunbio, China) or 0.5% CMC-Na via gavage once daily for consecutive 10 days. Tumor volume was measured every 3 days and calculated by caliper measurements of the width (W) and length (L) of each tumor using the following formula: V=(L×W^2^)/2. Mice were sacrificed and tumors were collected for further analysis. Ethical approvals for the animal experiments were obtained from the Ethics Committee of the First Affiliated Hospital of Zhengzhou University (Zhengzhou, China).

**RNA isolation and purification.** Total RNA was extracted from cells using Trizol reagent (Invitrogen) and mRNA enrichment was performed using Dynabeads mRNA Purification Kit (Invitrogen, 61,006) according to the manufacturer’s instructions with certain modifications. Briefly, RNA rebound and the DNase treatment of RNA were performed for the removal of contaminated rRNA and DNA, in the mRNA specimens, respectively. Finally, the concentration of the purified mRNA was determined using Qubit fluorometer (Invitrogen).

**Detection of m**^**5**^**C level.** The level of 5-methylcytosine (m^5^C) in mRNA was detected using MethylFlash 5-mC RNA Methylation ELISA Easy Kit (EpiGentek). 200 ng purified mRNA was bound to each well and incubated at 37 °C for 90 min. Then the wells were washed three times and incubated with 50 µL of the 5-mC Detection Complex Solution at room temperature for 50 min. Next, the wells were washed five times and incubated with 50 µL of Fluorescence Development Solution at room temperature for 4 min in darkness. The fluorescence was measured by EnVision microplate reader (PerkinElmer) within 10 min at 530ex/590em nm.

**Methylated RNA immunoprecipitation (MeRIP).** Purified mRNA was fragmented into around 100-nucleotide-long fragments using RNA Fragmentation Reagents (Invitrogen, AM8740). About 400 ng of fragmented mRNAs were mixed with 2.5 µg of anti-m^5^C antibody (Abcam, ab10805) in immunoprecipitation buffer and incubated by rotating at 25 °C for 1 h. The mixture was then immunoprecipitated by incubation with prewashed Protein A Magnetic Beads (Thermo Scientific, 10002D) at 4 °C for 5 h. After extensive washing, the bound RNA fragments were eluted from the beads by proteinase K digestion at 55 °C for 60 min. Finally, RNAs were isolated from the eluate by phenol-chloroform extraction and ethanol plus glycogen (Thermo Scientific, AM9516) precipitation for qRT-PCR analysis.

**RNA-seq.** RNA-seq libraries of the cell samples (20–30 ng mRNA) were constructed with the KAPA Stranded mRNA-Seq Kit (Illumina platform) following the manufacture’s protocol. Libraries sequencing were executed using paired-end mode on the Illumina HiSeq-PE150 instrument.

**RNA-BisSeq.** The process of RNA fragmentation and bisulfite conversion were performed as previously described with minor modifications [19]. Briefly, purified mRNA was mixed with Luciferase at a ratio of 300:1 and the Luciferase functioned as methylation conversion control. The libraries of the bisulfite-treatment samples were prepared using the KAPA Stranded mRNA-Seq Kit according to the manufacturer’s instructions (Illumina platform). Libraries sequencing were performed using paired-end mode by the Illumina HiSeq-PE150 system.

**RNA-Seq bioinformatics analyses.** Quality control of RNA-Seq data was processed by FastQC. Adaptors were trimmed using cutadapt and low-quality bases were removed with Trimmomatic. The clean raw reads were mapped to the hg38 genome with the Ensembl 78 genome annotation with default parameters by hisat2 and Htseq-count was selected for counting the number of reads mapped to each Ensembl gene with the following parameters: -m union -s no. DESeq2 was chosen for analyzing the genes expression by calculating the TPM of the gene in each sample. Differentially expressed genes were assigned with cut-off |FoldChange| ≥ 1.2 and adjusted p-value < 0.05.

**RNA-BisSeq bioinformatics analyses.** RNA-BisSeq reads for each sample were extensively filtered and processed by FastQC which was similar with “RNA-Seq bioinformatics analyses” section described. Clean reads of Bis-treated libraries were mapped to human genome (hg38) using meRanGs, a splice-aware RNA-BSseq alignment tool available with meRanTK. The processed reads with lengths greater than 35 nt were defined as clean reads. The sample with C to T conversion rates > 99% were filtered for further analysis. The m^5^C sites within the genome were extracted by meRanCall with the following criteria: -mBQ 20 -mr 0.1. In order to identify the credible m^5^C sites, only candidate m^5^C sites with coverage depth (methylated C number + nonmethylated C number) ≥ 20, methylated cytosine depth ≥ 3 and m^5^C methylation level ≥ 0.1 in at least half replicates in any one condition were kept and used for differential methylation analysis after calling. The m^5^C sites were annotated using BEDTools intersectBed. The top hits with |Methylation level A – Methylation level B| > 0.05 were considered as differentially meythylated m^5^C sites. Cluster profiler was used to analyze the significantly differential genes for GO and KEGG pathway enrichment. The GO terms and KEGG pathways with adjusted p-value < 0.05 were considered as the significantly differential pathways.

### Statistical analysis

Data were presented as the means ± standard deviation (SD), unless stated otherwise. Two-tailed Student’s t test was used to compare means between groups as indicated. The OS and RFS were assessed using the Kaplan-Meier analysis and the p values were calculated using the two-sided log-rank test. *p < 0.05 was considered significant.

## Electronic supplementary material

Below is the link to the electronic supplementary material.

Supplementary Material 1

Supplementary Material 2

Supplementary Material 3

Supplementary Material 4

Supplementary Material 5

Supplementary Material 6

Supplementary Material 7

Supplementary Material 8

Supplementary Material 9

## Data Availability

The raw data of RNA-BisSeq and RNA-seq reported in this paper have been deposited in the Genome Sequence Archive (GSA: HRA002603 linked to the BioProject with accession Number PRJCA010074).

## Abbreviations

ALYREF: Aly/REF export factor
CDS: coding sequence
DNMT2: DNA methyltransferase 2
GEO: Gene Expression Omnibus
m^5^C: 5-methylcytosine
m^6^A: N6-methyladenosine
MeRIP: Methylated RNA immunoprecipitation
NSCLC: non-small-cell lung cancer
NSUN: NOP2/Sun domain
IHC: immunohistochemistry
IF: immunofluorescence
QSOX1: quiescin sulfhydryl oxidase 1
qRT-PCR: quantitative real-time PCR
RIP: RNA immunoprecipitation
RNA-seq: RNA-sequencing
RNA-BisSeq: RNA bisulfite sequencing
SCLC: small cell lung cancer
TKI: tyrosine kinase inhibitor
TCGA: the Cancer Genome Atlas
TET2: tet methylcytosine dioxygenase 2
YBX1: Y -box binding protein 1
