# Supplementary figures and images for: Aberrant m5C hypermethylation mediates intrinsic resistance to gefitinib through NSUN2/YBX1/QSOX1 axis in EGFR-mutant non-small-cell lung cancer
Source: Mol Cancer. 2023 May 9;22:81. doi: 10.1186/s12943-023-01780-4 (PMC10169458; doi:10.1186/s12943-023-01780-4)

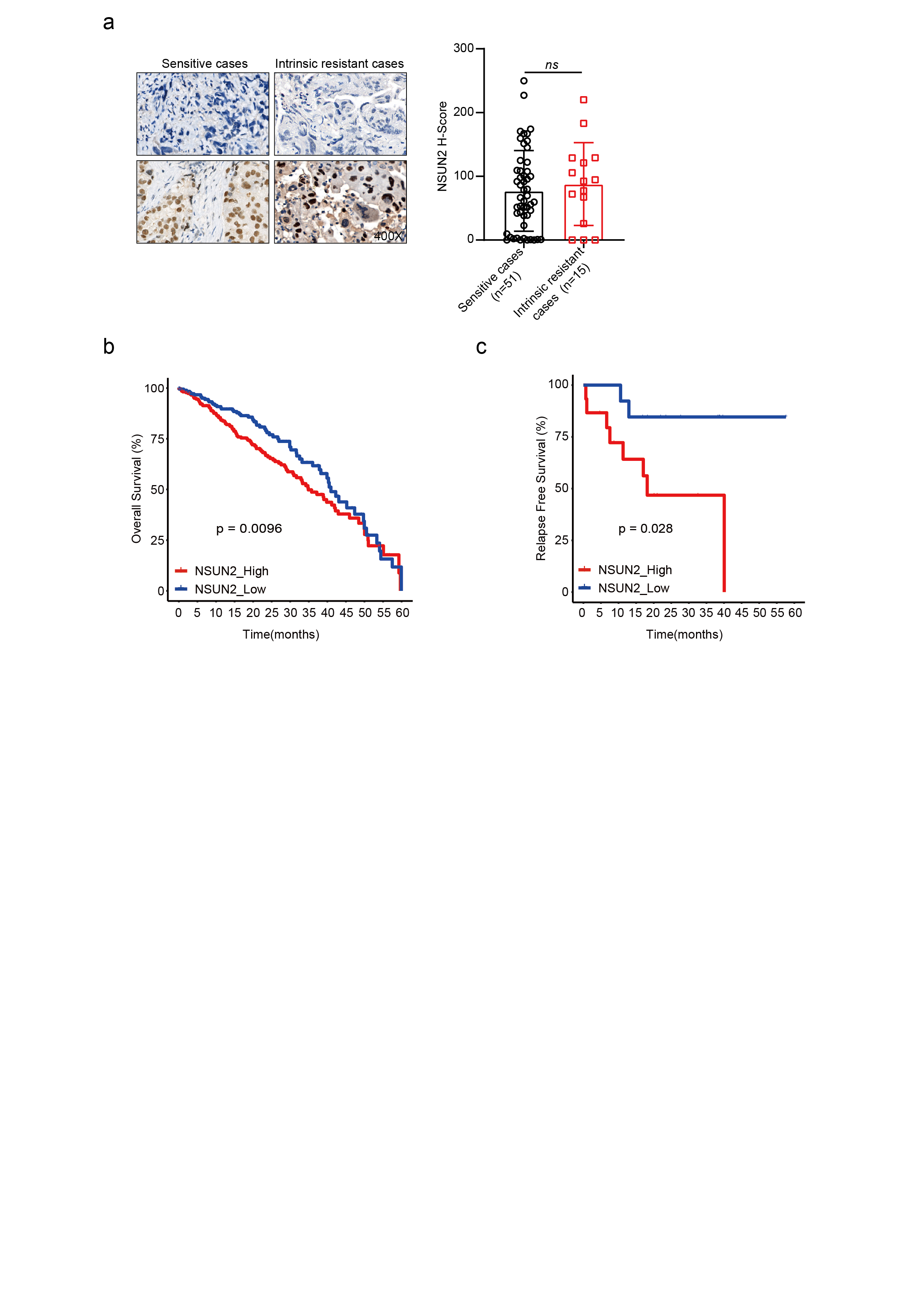

Supplement: Supplementary file 1 — Supplementary Material 1 [file 12943_2023_1780_MOESM1_ESM.png]

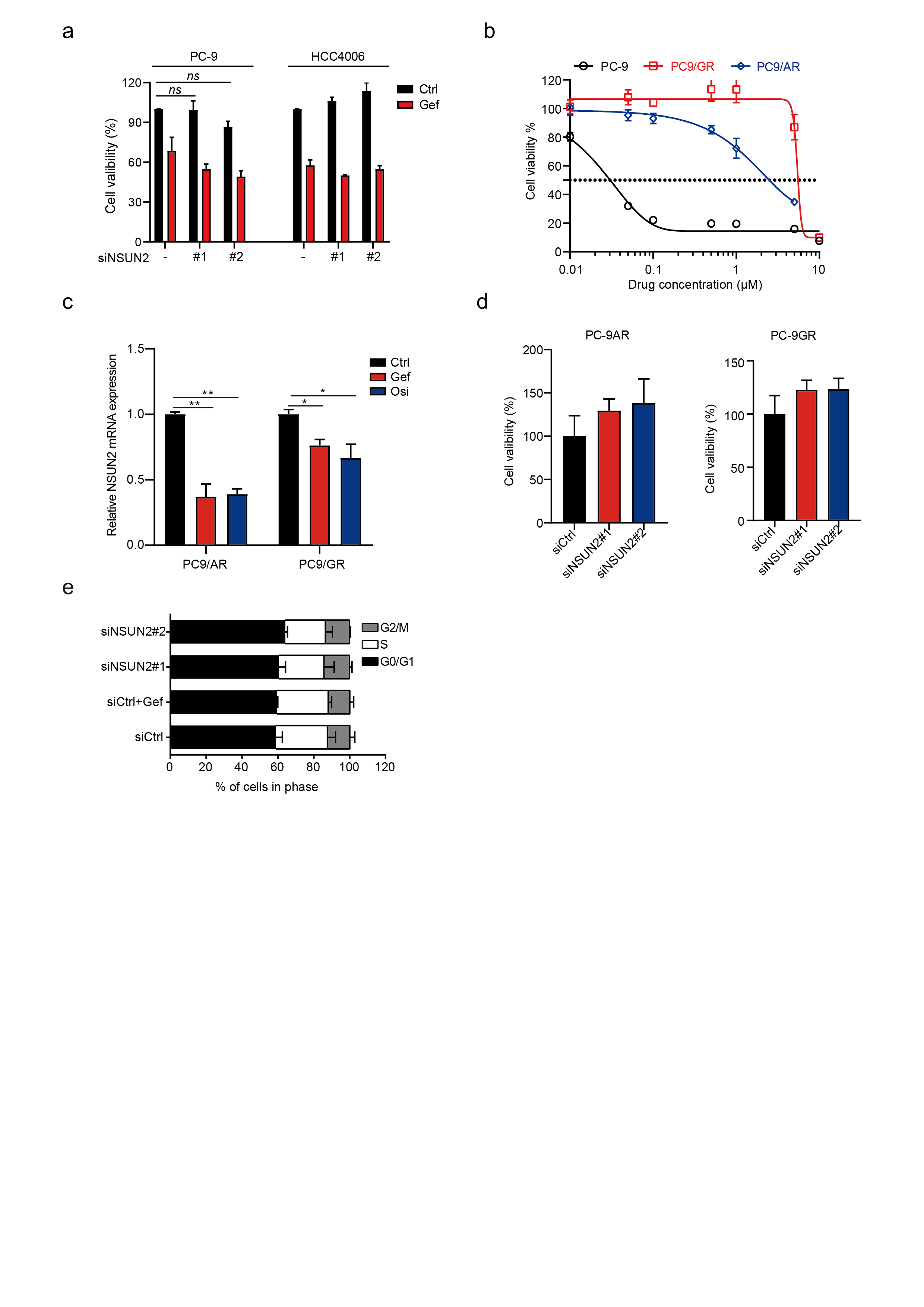

Supplement: Supplementary file 2 — Supplementary Material 2 [file 12943_2023_1780_MOESM2_ESM.png]

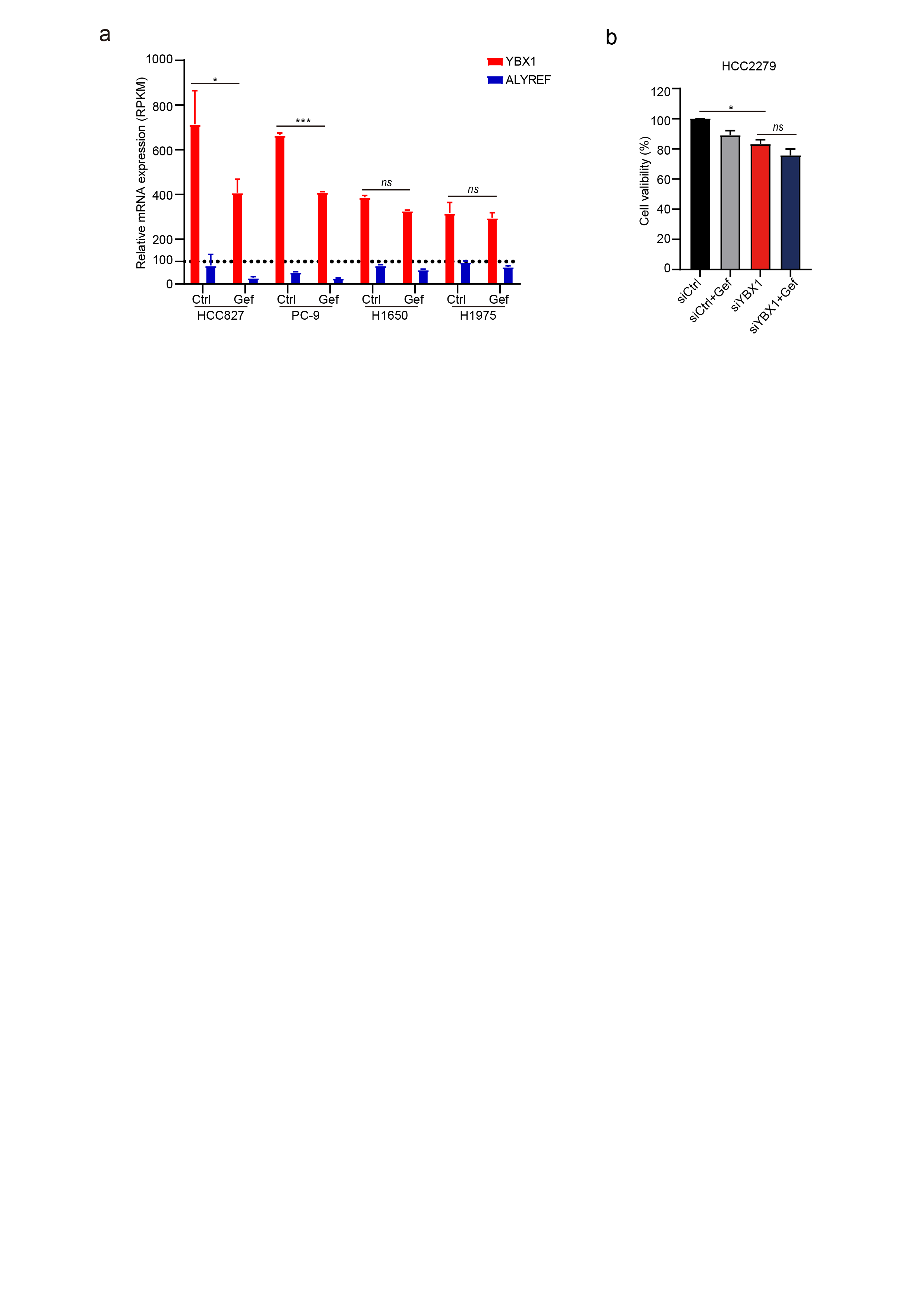

Supplement: Supplementary file 3 — Supplementary Material 3 [file 12943_2023_1780_MOESM3_ESM.png]

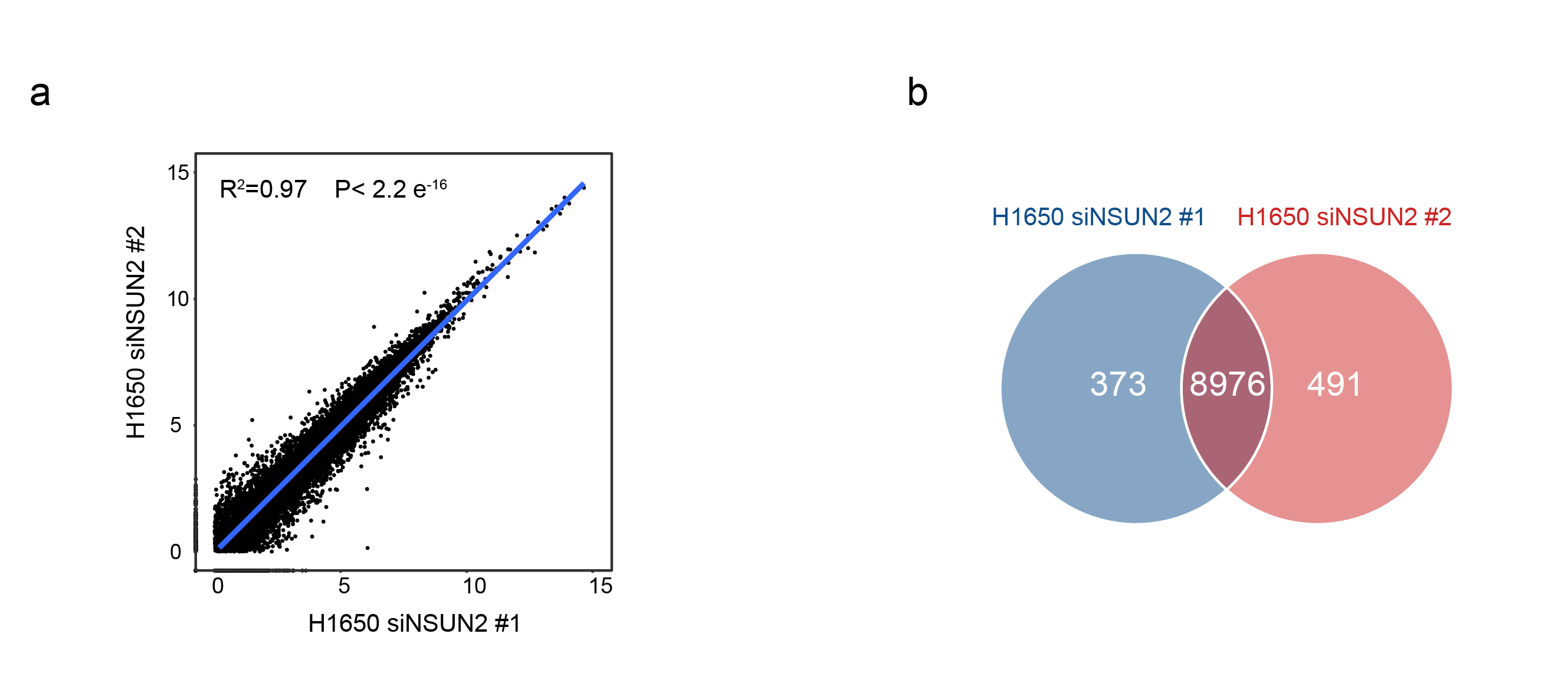

Supplement: Supplementary file 4 — Supplementary Material 4 [file 12943_2023_1780_MOESM4_ESM.png]

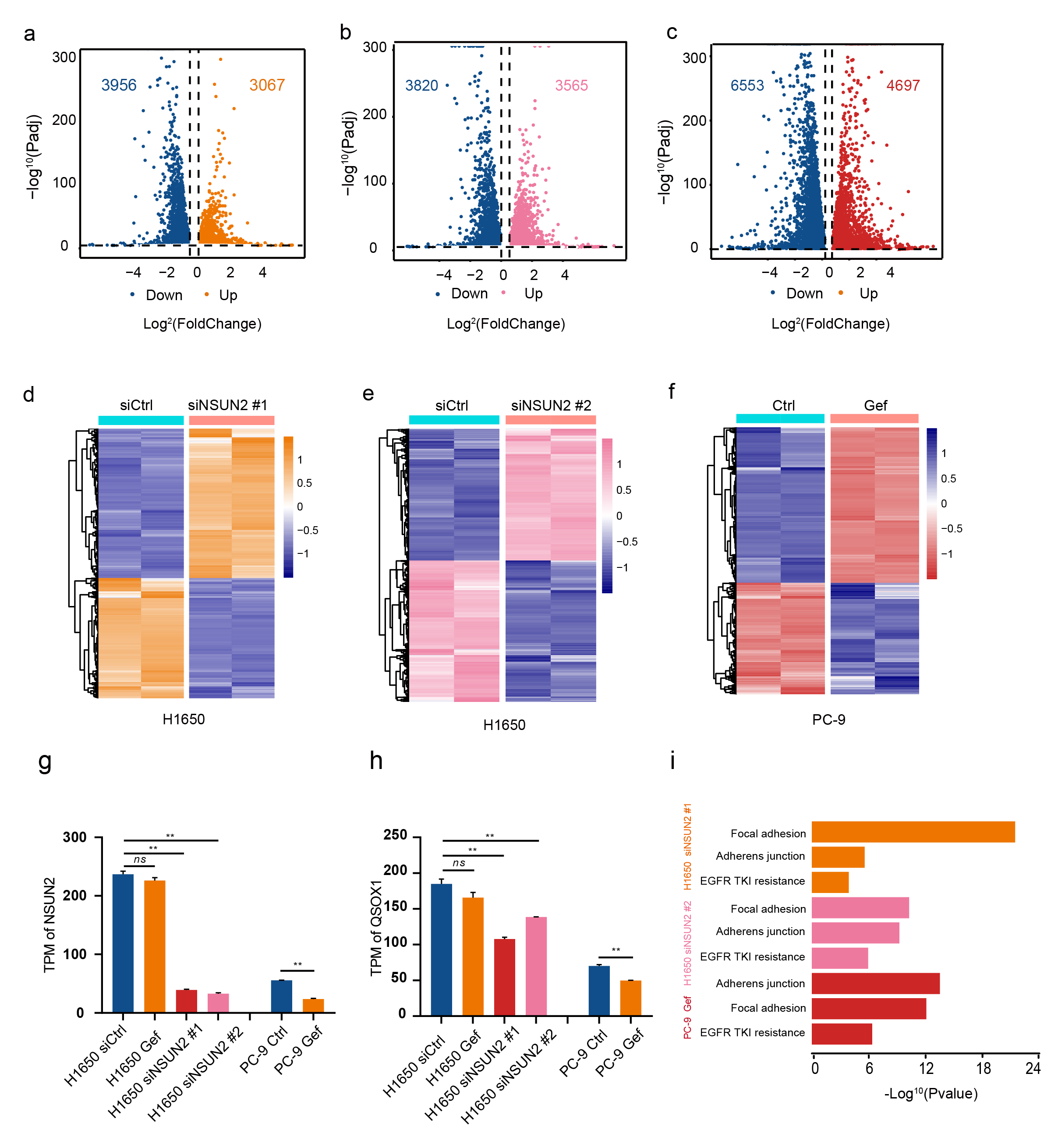

Supplement: Supplementary file 5 — Supplementary Material 5 [file 12943_2023_1780_MOESM5_ESM.png]

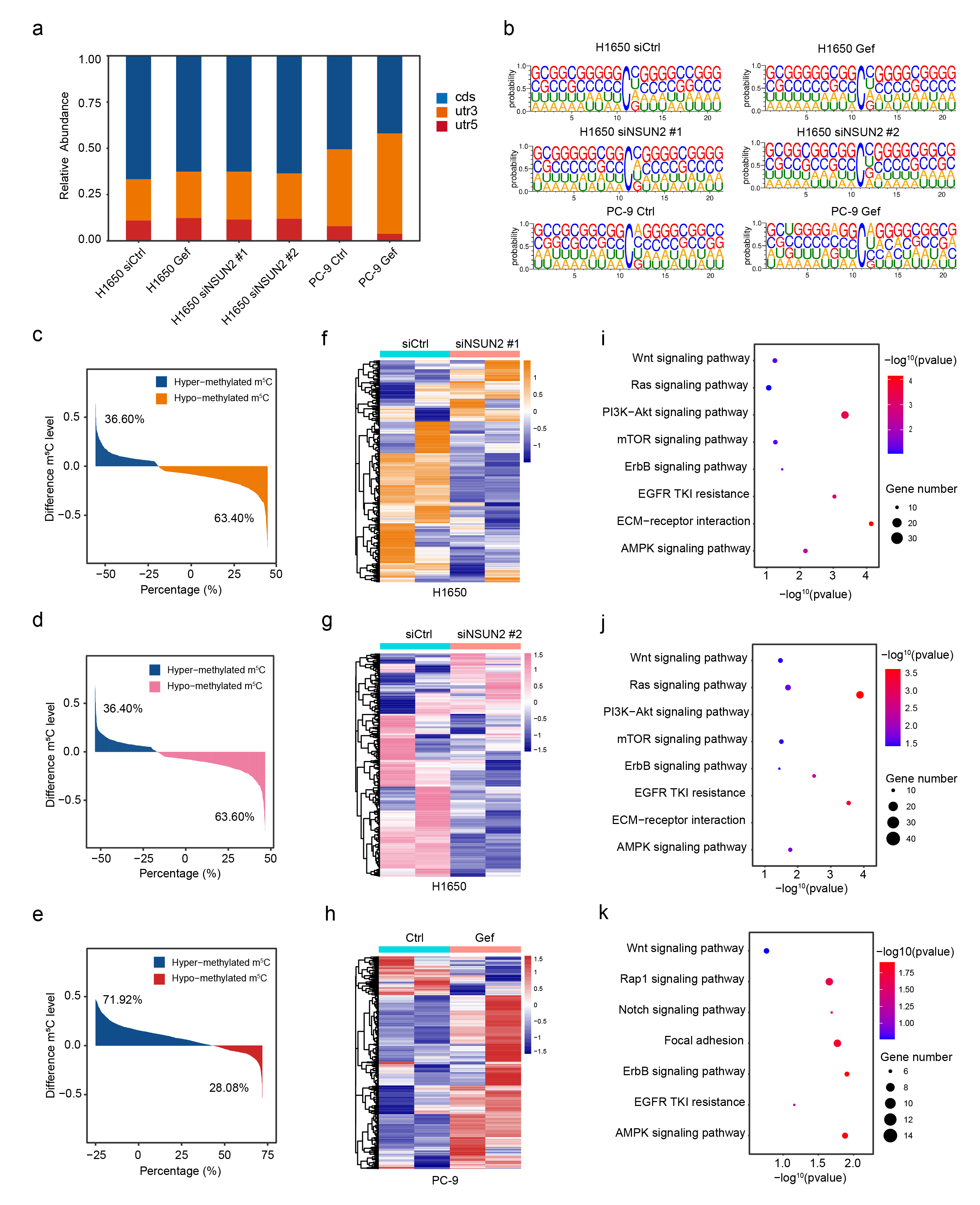

Supplement: Supplementary file 6 — Supplementary Material 6 [file 12943_2023_1780_MOESM6_ESM.png]

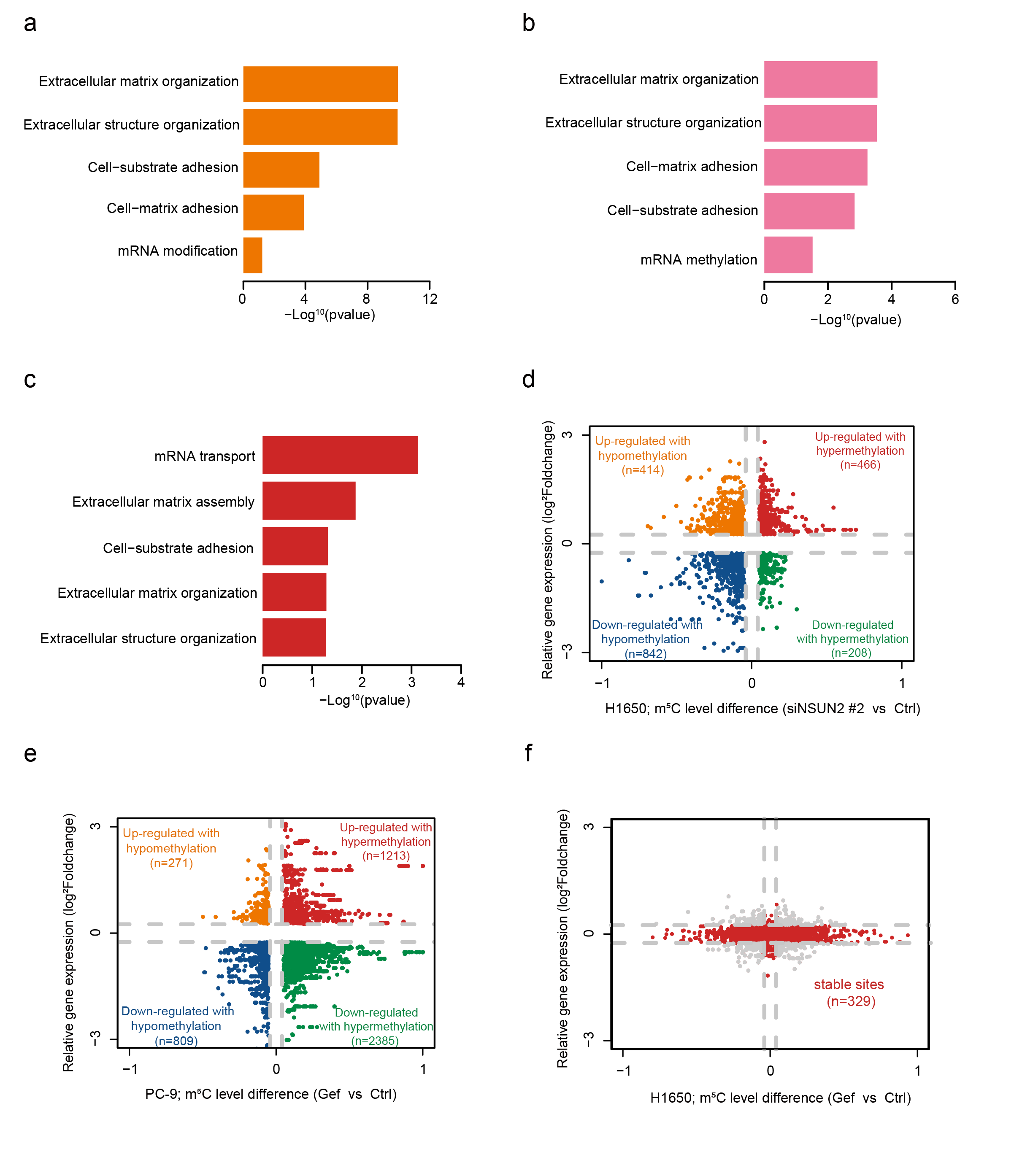

Supplement: Supplementary file 7 — Supplementary Material 7 [file 12943_2023_1780_MOESM7_ESM.png]

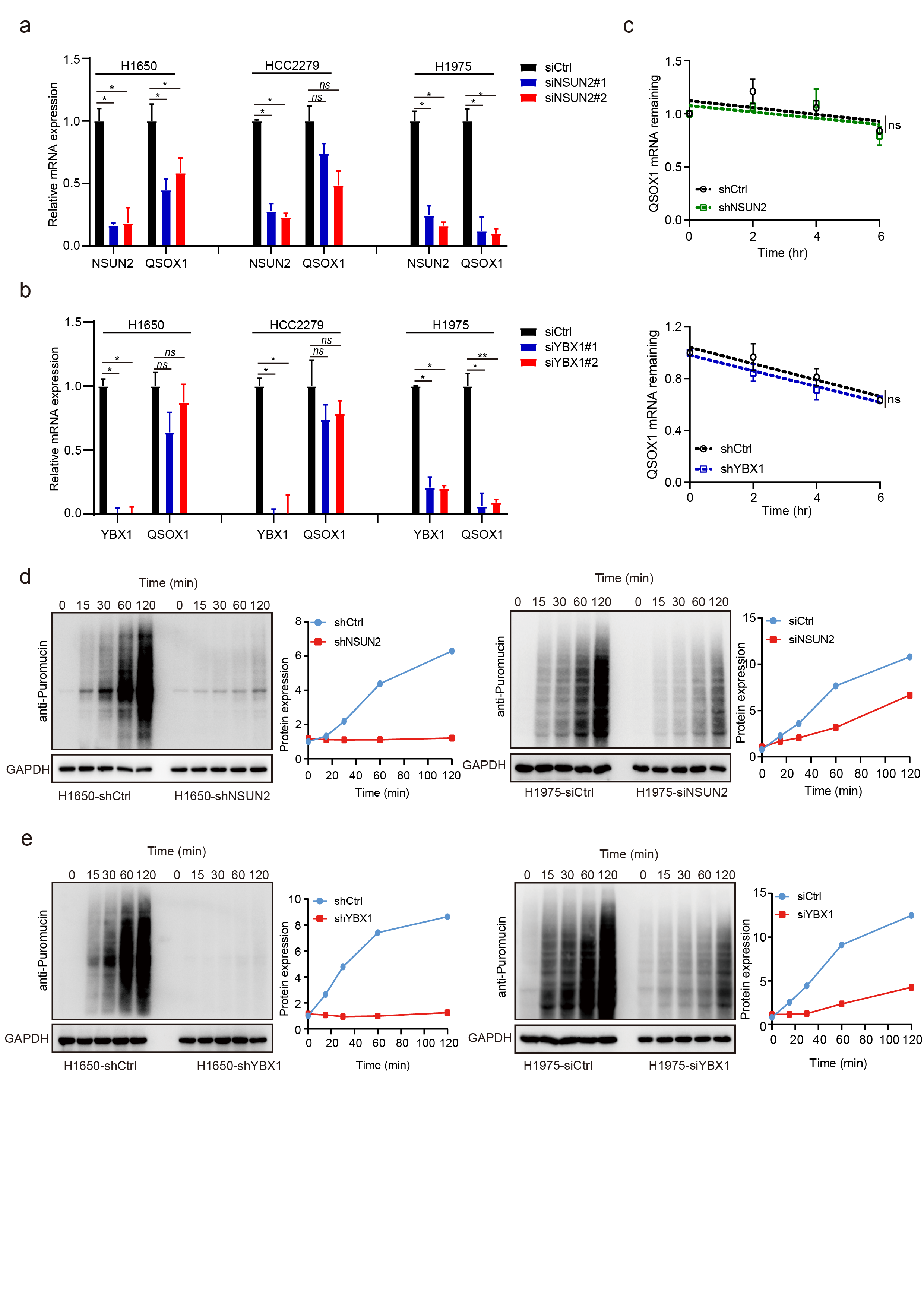

Supplement: Supplementary file 8 — Supplementary Material 8 [file 12943_2023_1780_MOESM8_ESM.png]

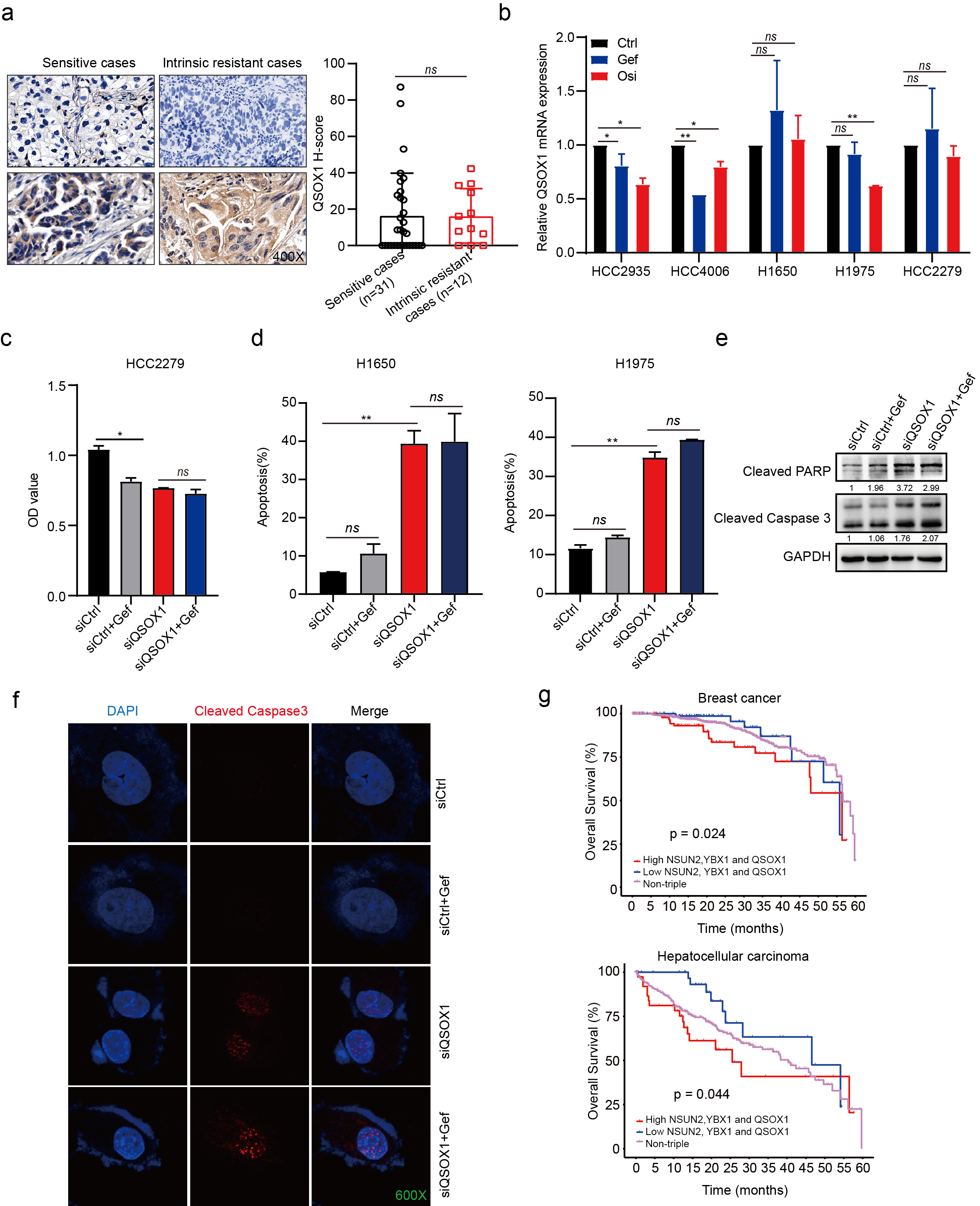

Supplement: Supplementary file 9 — Supplementary Material 9 [file 12943_2023_1780_MOESM9_ESM.png]
